# Therapeutic potential of quercetin in depressive symptoms: a systematic review and meta-analysis of preclinical studies

**DOI:** 10.3389/fphar.2025.1598053

**Published:** 2025-07-08

**Authors:** Yang Yang, Yingshi Zhang, Lixin Chen, Ze Li, Qingchun Zhao

**Affiliations:** ^1^ School of Life Sciences and Biopharmaceuticals, Shenyang Pharmaceutical University, Shenyang, Liaoning, China; ^2^ Department of Pharmacy, Jinqiu Hospital of Liaoning Province, Shenyang, Liaoning, China; ^3^ Department of Pharmacy, General Hospital of Northern Theater Command, Shenyang, Liaoning, China

**Keywords:** quercetin, depression, systematic review, meta-analysis, preclinical studies

## Abstract

**Background:**

Depression is a common and severe mental disorder. Quercetin, a natural flavonoid compound, has been shown in several studies through animal experiments to improve depressive symptoms, demonstrating significant antidepressant potential.

**Objective:**

This study represents the first preclinical meta-analysis on quercetin and depression, aiming to systematically evaluate the antidepressant effects of quercetin in animal studies. Methods: This study conducted a systematic search of the PubMed, EMBASE, Cochrane Library, and Web of Science electronic databases, with the search period covering from the inception of the databases to January 2025. Subsequently, the SYRCLE risk of bias assessment tool was used for quality evaluation, and data analysis was performed using RevMan 5.4 software.

**Results:**

This systematic review included 52 animal studies for random-effects meta-analysis. The results indicated that, compared to the control group, quercetin significantly reduced the immobility time in the forced swimming test and tail suspension test, as well as the time spent in the closed arms of the elevated plus maze. Simultaneously, it increased sucrose preference, swimming time in the forced swimming test, total distance traveled in the open field test, time spent in the central area, and the number of entries into the central area. In the elevated plus maze test, quercetin also increased the time spent in the open arms and the number of entries into the open arms. However, it did not produce a significant effect on the number of standing episodes in the open field test. Moreover, quercetin increased the levels of glutathione (GSH), superoxide dismutase (SOD), catalase (CAT), and brain-derived neurotrophic factor (BDNF), while reducing the levels of malondialdehyde (MDA), tumor necrosis factor-α (TNF-α), interleukin-1β (IL-1β), interleukin-6 (IL-6), and corticosterone (CORT).

**Conclusion:**

This meta-analysis indicates that quercetin significantly improves depressive symptoms. However, further high-quality studies are needed to explore the role of quercetin in antidepressant research.

**Systematic Review Registration:**

http://inplasy.com/, Identifier: INPLASY202530047.

## Graphical Abstract

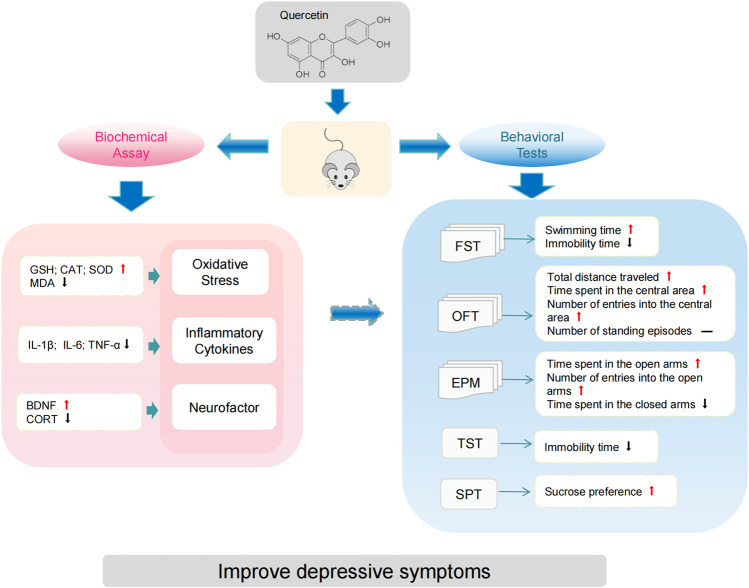

## 1 Introduction

Depression is a common and highly recurrent mental disorder globally, characterized by persistent low mood, anxiety, anhedonia, and cognitive impairments (Burcusa and Iacono, 2007; Malhi and Mann, 2018). According to reports by the World Health Organization (WHO), over 300 million people worldwide suffer from depression. More than 700 000 people die due to suicide every year (World Health Organization, 2020). It is expected that by 2030, depression will become the leading cause of disability worldwide (Chen et al., 2024), placing a significant psychological and economic burden on individuals, families, and society. In recent years, treatment methods for depression have continuously evolved, including pharmacotherapy, psychotherapy, neuromodulation technologies, AI-assisted diagnosis and treatment, and lifestyle interventions (Sampogna et al., 2024). However, pharmacological intervention remains the cornerstone of depression treatment, with most currently used antidepressants exhibiting limitations such as a single mechanism of action, slow onset, and significant side effects (Pannu et al., 2021). Therefore, there is an urgent need to develop safe and effective antidepressants that target multiple pathways and mechanisms.

Quercetin (3,3′,4′,5,7-hydroxyflavone) is a natural flavonoid compound widely found in plants such as apples, onions, broccoli, wine, green tea, and ginkgo (Williamson and Manach, 2005; Mirza et al., 2023). Many studies have shown that quercetin exhibits various biological activities, including antioxidant (Xu et al., 2019), anti-inflammatory (Wu et al., 2024), and anticancer properties (Maleki et al., 2021), and can protect the nervous system (Cardozo et al., 2021; Fideles et al., 2023) as well as improve cognitive function (Ebrahimpour et al., 2020). In recent years, research on quercetin’s antidepressant effects has been gradually increasing. Current studies indicate that quercetin enhances the expression of BDNF in the prefrontal cortex and hippocampus of mice, improving anxiety, depression, and cognitive deficits induced by psychosocial stress (Ugwu et al., 2022). Other studies suggest that quercetin regulates Acetyl-H3K9 and inhibits astrocyte ferroptosis, significantly improving depressive-like behaviors in a perimenopausal depression rat model (Wang et al., 2024c). Furthermore, Ge et al. discovered that quercetin reduces apoptosis in the hippocampus and prefrontal cortex of chronic unpredictable stress (CUS) model mice, upregulates Nrf2 protein expression, and increases the phosphorylation levels of ERK and CREB, improving depressive behaviors in mice, similar to the effects of the antidepressant fluoxetine (Ge et al., 2024).

Despite increasing evidence confirming the effectiveness of quercetin in treating depression (Chen et al., 2022), some controversial results remain in the published studies. To clarify the clinical indications of quercetin for depression, a comprehensive and scientific evaluation of animal experimental studies is crucial. Therefore, we have summarized the existing evidence and, for the first time, conducted a meta-analysis of preclinical studies on quercetin’s antidepressant effects, which may provide important clues for future clinical research.

## 2 Materials and methods

This systematic review and meta-analysis were conducted in accordance with the PRISMA guidelines (Page et al., 2021) and the Cochrane Collaboration’s principles. To avoid duplication with ongoing systematic reviews, we first searched for similar reviews on the INPLASY website and subsequently registered our study (registration number: INPLASY202530047).

### 2.1 Search strategy

We systematically searched four electronic databases (PubMed, EMBASE, Cochrane Library, Web of Science) for data from the inception of the databases through January 2025. The search strategy utilized terms related to quercetin and depression to identify preclinical studies assessing the impact of quercetin on depression. The search algorithm employed only terms relevant to the topic of interest and filtered unique keywords to database (Reshma et al., 2024). The detailed search strategy is provided in Table 1 (using PubMed as an example).

**TABLE 1:** Search strategy on PubMed.

#1	“ depression “ [MeSH] OR” depressive disorder “ [MeSH]
#2	((((((((((((Depression [Title/Abstract]) OR (Depressive Disorder [Title/Abstract])) OR (Depressive Symptoms [Title/Abstract])) OR (Depressive Symptom [Title/Abstract])) OR (Symptom, Depressive [Title/Abstract])) OR (Emotional Depression [Title/Abstract])) OR (Depression, Emotional [Title/Abstract])) OR (Melancholia [Title/Abstract])) OR (Depressive Syndrome [Title/Abstract])) OR (major depression [Title/Abstract])) OR (refractory depression [Title/Abstract])) OR (anxiety disorders [Title/Abstract])) OR (affective disorders [Title/Abstract])
#3	#1 OR #2
#4	“ Quercetin “ [MeSH]
#5	(((((Quercetin [Title/Abstract]) OR (Pentahydroxyflavone [Title/Abstract])) OR (Dikvertin [Title/Abstract])) OR (Quercetins [Title/Abstract])) OR (Quercetol [Title/Abstract])) OR (Sophoretin [Title/Abstract])
#6	#4 OR #5
#7	#3 AND #6

### 2.2 Inclusion and exclusion criteria

Based on the PICOS principle, the studies included in this review adhered to the following criteria: (P) Population: Animal studies, with preparation requiring ethical approval, and no restrictions on species, gender, age, or weight of the animals; (I) Intervention: Studies involving quercetin treatment, either alone or in combination, with no restrictions on the route of administration, duration, dosage, or formulation; (C) Comparison: Control groups with either blank controls or standard treatments; (O) Outcome: Primary outcome measures included the forced swimming test (FST), tail suspension test (TST), sucrose preference test (SPT), open field test (OFT), and elevated plus maze (EPM); secondary outcome measures included corticosterone (CORT), brain-derived neurotrophic factor (BDNF), catalase (CAT), malondialdehyde (MDA), superoxide dismutase (SOD), glutathione (GSH), interleukin-1β (IL-1β), interleukin-6 (IL-6), and tumor necrosis factor-α (TNF-α); (S) Study Design: Animal experiments.

The exclusion criteria were as follows: (1) Review articles, case reports, editorials/letters, patents, abstracts, and other informal journals; (2) *In vitro* studies, computer simulation studies, and all clinical trials; (3) Republished and irrelevant literature; (4) Studies on quercetin derivatives; (5) Experimental studies lacking a control group; (6) Studies with missing original articles or incomplete original data; (7) Studies where outcome data cannot be extracted or merged.

### 2.3 Research selection

The literature was screened and excluded using EndNote reference management software. Two researchers independently reviewed the titles and abstracts of the studies, excluded those that did not meet the inclusion criteria, and determined which studies to include. The remaining studies were then read in full by both researchers to further confirm their inclusion. Throughout the screening process, both researchers worked independently, and the final list of included studies was compared. If the studies matched, they were included; if there was a discrepancy, a third researcher discussed and resolved the differences.

### 2.4 Data extraction

The following information was independently extracted from the included studies by two researchers: (1) First author’s name and publication year; (2) Animal characteristics described in the intervention and control groups, including animal model, species, sex, and the number of animals per group; (3) Treatment information, including the drug dosage, route of administration, and duration of the intervention; (4) Outcome measures: Two researchers independently extracted data from each study, initially attempting to extract numerical data from tables or text. If these were not reported, quantitative data were extracted from graphs using Engauge Digitizer.

### 2.5 Quality assessment

Two researchers independently assessed the risk of bias in each included study using the SYRCLE risk of bias assessment tool (Hooijmans et al., 2014), specifically analyzing the following types of bias: selection bias (random sequence generation, baseline characteristics, allocation concealment), performance bias (random housing, blinding), detection bias (random outcome assessment, blinding), attrition bias (incomplete outcome data), reporting bias (selective reporting), and other biases. Each type of bias was classified as high risk, low risk, or unclear. In the event of a discrepancy, the issue was resolved through discussion with a third researcher.

### 2.6 Data analysis

In this meta-analysis, the outcome measures were continuous data. Due to differences in animal species and models, standardized mean differences (SMD) and 95% confidence intervals (CIs) were used for analysis. The heterogeneity of the data was assessed using the *I* ^*2*^ statistic. When *I* ^*2*^ <50% and p > 0.05, low heterogeneity was assumed, and a fixed-effect model was used; otherwise, a random-effects model was applied. Sensitivity analysis was performed when more than 10 studies were included in an outcome measure, and funnel plots, along with Begg’s and Egger’s tests, were used to assess publication bias. A p-value <0.05 was considered statistically significant. All data analyses were performed using Review Manager 5.4 and STATA 15.1 software.

## 3 Results

### 3.1 Study selection

**FIGURE 1:**
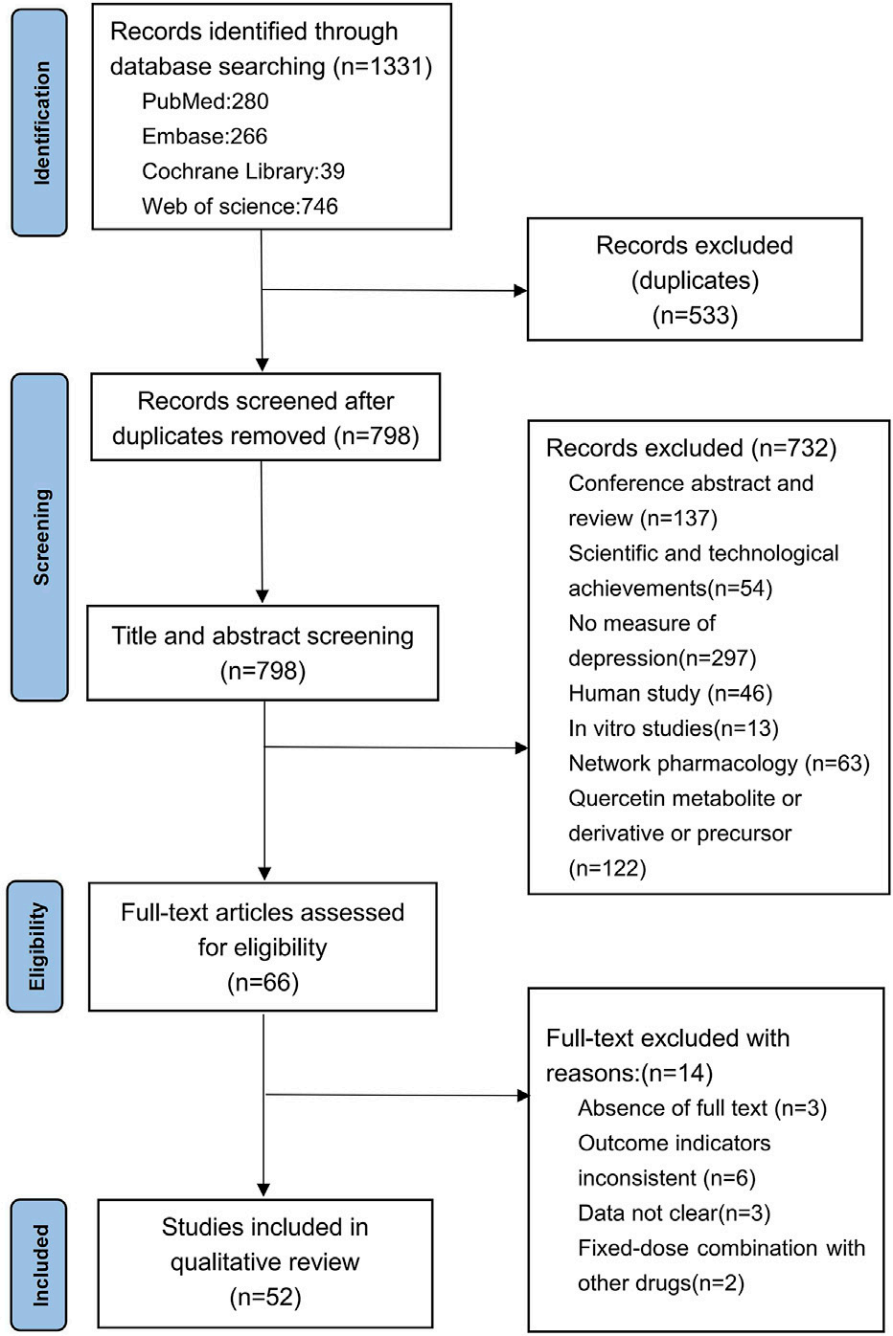
Flow chart of the literature search.

Based on the predefined search strategy, a total of 1,331 articles were retrieved. After excluding 533 duplicates, 798 articles remained. By reviewing the titles and abstracts, we further excluded conference abstracts and reviews (n = 137), technological achievements (n = 54), studies not measuring depression (n = 297), human studies (n = 46), *in vitro* studies (n = 13), network pharmacology studies (n = 63), and studies on quercetin metabolites, derivatives, or precursors (n = 122). Finally, 66 articles were selected for full-text screening. Afterward, we excluded studies with no full text (n = 3), studies with inconsistent outcome measures (n = 6), studies with unclear data (n = 3), and studies involving fixed-dose combinations with other drugs (n = 2), resulting in the inclusion of 52 articles (Anjaneyulu et al., 2003; Sah et al., 2011; Liu et al., 2013; Rinwa and Kumar, 2013; Jain and Gangshettiwar, 2014; Merzoug et al., 2014; Holzmann et al., 2015; Mehta et al., 2017; Rebai et al., 2017; Singh et al., 2017; Quraishi et al., 2018; Samad et al., 2018; Anggreini et al., 2019; Fang et al., 2019; Khan et al., 2019; Sriram and Ravichandra, 2019; Toumi et al., 2019; Boudia et al., 2020; Donoso et al., 2020; Sadighparvar et al., 2020; Ahin et al., 2020; Zhang et al., 2020; Bicca et al., 2021; Bin-Jaliah, 2021; Eduviere et al., 2021; Guan T. et al., 2021; Guan Y. et al., 2021; Ma et al., 2021; Madiha et al., 2021; Wang et al., 2021; Tan et al., 2022; Ugwu et al., 2022; Yang et al., 2022; Adeoluwa et al., 2023; Balasubramanian et al., 2023; Ge et al., 2023; Jia et al., 2023; Adeoluwa et al., 2024; Bappi et al., 2024; Du et al., 2024; Ge et al., 2024; Kore et al., 2024; Li B. et al., 2024; Li Y. et al., 2024; Makhdoomi et al., 2024; Su et al., 2024; Tavakol et al., 2024; Wang et al., 2024c; Wang et al., 2024a; Wang et al., 2024b; Zhu et al., 2024; Hou et al., 2025) for the comprehensive analysis. The detailed literature search and screening process is shown in Figure 1.

### 3.2 Characteristics and quality of included studies

A total of 52 studies included in this meta-analysis were published between 2003 and 2025, and their main characteristics are summarized in Table 2. Of these, 21 studies were conducted in China, 11 in India, 4 in Nigeria and Algeria, 3 in Iran, 2 in Brazil and Pakistan, and one each in Indonesia, Bangladesh, Saudi Arabia, Ireland, and Turkey. Twenty-five studies induced depression in animals through stress, 15 through chemical induction, 5 through surgical induction, and 1 through genetic knockout. The sample size of each study ranged from 3 to 24 animals. Of the included studies, 28 used mice (weighing 10–30 g), and 24 used rats (weighing 80–300 g). Except for 10 studies, the remaining all used male animals. The quercetin dosage ranged from 50 μg/kg to 2 g/kg. Twenty-one studies administered quercetin orally, 19 via gavage, and the remaining 12 via intraperitoneal injection. The duration of quercetin supplementation ranged from 0.5 h to 12 weeks. Among the 52 included studies, the baseline characteristics were evenly distributed between the experimental and control groups in all studies. Random sequence generation was reported in 41 studies (78.8%), but none of the studies mentioned allocation concealment or blinding of animal breeders and researchers. Seven studies performed blinded outcome assessment. In addition, all studies reported the completeness of outcome data, with all animals undergoing the same assessments during the experiment, and no selective reporting was detected. Overall, the quality of the included studies primarily ranged from moderate to low risk of bias. There was a high number of “unclear risk” or “high risk” bias sources in allocation concealment and blinding, but most studies showed a low risk of bias in random sequence generation, outcome assessment, and other biases, providing a certain level of reliability to the meta-analysis results. However, caution should still be taken when interpreting and applying these results due to the potential impact of these biases. The detailed information on the quality assessment of the literature is presented in Table 3.

**TABLE 2:** Characteristics of the 52 studies included in the meta-analysis.

First author	Publication year	Country	Species	Weight	Sex	Numbers in each group (QE/Ctrl)	Model	Experimental group	Dose of quercetin	Control group	Method of administration	Administration time	Outcome measures
Muragundla Anjaneyulu	2003	India	Laka mice	20–30 g	male	6/6	Streptozotocin-Induced Diabetic	quercetin + Diabetic	100 mg/kg	vehicle + Diabetic	i.p	60min	A
Sangeeta Pilkhwal Sah	2011	India	Wistar albino rat	150–200 g	—	6/6	LPS	quercetin + LPS	25 mg/kg	vehicle + LPS	i.p	2w	BEFGKL
Jianxiang Liu	2013	China	ICR mice	21–25 g	male	15/15	—	quercetin	20 mg/kg	blank control	i.g	1 h	C
Puneet Rinwa	2013	India	Wistar rat	250–300 g	male	6/6	Olfactory bulbectomy (OBX)	quercetin + OBX	80 mg/kg	OBX	p.o	2w	ABFGJKLMN
Dilpesh Jain	2014	India	Wistar rat	200–250 g	male	6/6	3-nitropropionic acid (3-NP)-induced Huntington’s disease	quercetin+3-NP	50 mg/kg	3-NP	p.o	14 d	A
Sameha Merzoug	2014	Algeria	Wistar rat	—	male	5/5	Adriamycin (ADR)	quercetin + ADR	60 mg/kg	ADR	i.p	24 h	ABEJKL
Iandra Holzmann	2015	Brazil	Swiss mice	25–30 g	female	6/6	Olfactory bulbectomy (OB)	quercetin + OB	25 mg/kg	vehicle + OB	p.o	14 d	ABCKN
Vineet Mehta	2017	India	Swiss albino mice	20–25 g	—	8/8	CUS	quercetin + CUS	30 mg/kg	CUS	p.o	21 d	BDELM
Redouane Rebai	2017	Algeria	Wistar rat	180–210 g	male	7/6	Streptozotocin-Induced Diabetic	quercetin + Diabetic	10 mg/kg	vehicle + Diabetic	i.p	4w	AB
Tanveer Singh	2017	India	Swiss albino mice	22–28 g	male	6/6	Pentylenetetrazole induced kindling	quercetin + levetiracetam + kindled	40 mg/kg	levetiracetam + kindled	p.o	15 d	CDJ
Mustajab Quraishi	2018	India	Wistar rat	180–220 g	male	6/6	CUMS	quercetin + CUMS	50 mg/kg	saline + CUMS	p.o	1w	ABDJ
Noreen Samad	2018	Pakistan	Albino Wistar mice	20 ± 5 g	male	6/6	2 h immobilization stress	quercetin + stress	20 mg/kg	vehicle + stress	i.p	14 d	AELMN
Khadeeja Khan	2019	India	Swiss albino mice	25–30 g	male	5/5	CUMS	quercetin + CUMS	25 mg/kg	saline + CUMS	p.o	4w	ABCFGKMN
Putri Anggreini	2019	Indonesia	ICR mice	25–30 g	male	6/6	Predatory stress	quercetin + Predatory stress	50 mg/kg	Predatory stress	i.p	3 d	CE
SRIRAM BS	2019	India	Mice	25–30 g	male	6/6	Monosodium glutamate (MSG)	quercetin + MSG	100 mg/kg	MSG	p.o	13 d	FI
Mohamed Lamine Toumi	2019	Algeria	Wistar rat	255 ± 5 g	male	7/7	Alloxan Induced Diabetic	quercetin + Diabetic	100 mg/kg	vehicle + Diabetic	p.o	24 h	ABE
Fang Ke	2019	China	SD rat	—	male	3/3	LPS	quercetin + LPS	40 mg/kg	LPS	i.g	14 d	ABDFI
Fella Boudiaf	2020	Algeria	Wistar rat	210 ± 20 g	—	6/6	—	quercetin	5 mg/kg	blank control	i.g	7 d	B
Francisco Donoso	2020	Ireland	SD rat	250–300 g	female	10/12	Maternal separation (MS)	quercetin + MS	20 mg/kg	MS	p.o	8w	BEIJ
Jiajia Zhang	2020	China	C57BL/6J mice	—	male	24/22	Chronic social defeat stress (CSDS)	quercetin + CSDS	2 g/kg	CSDS	p.o	54 d	BCDE
Shirin Sadighparvar	2020	Iran	Wistar rat	90 ± 10 g	male	6/6	1,2-dimethyhydrazine (DMH)-induced colorectal cancer	quercetin + exercise + DMH	50 mg/kg	exercise + DMH	i.g	12w	ABGHI
Tuğçe DemirtaşŞahin	2020	Turkey	Wistar albino rat	250–300 g	male	8/8	CUMS	quercetin + CUMS	30 mg/kg	vehicle + CUMS	i.p	5w	ADJKLMN
Ismaeel Bin-Jaliah	2021	Saudi Arabia	Wistar rat	150–200 g	male	6/6	CUS	quercetin + CUS	50 mg/kg	saline + CUS	i.p	3w	LN
Anthony Taghogho Eduvière	2021	Nigeria	Albino Swiss mice	22.0 ± 2.0 g	male	6/6	72 h active sleep disruption	quercetin + sleep-deprived	50 mg/kg	vehicle + sleep-deprived	p.o	7 d	CMN
Tong Guan	2021	China	SD rat	190–220 g	male	10/10	CUMS	quercetin + CUMS	50 mg/kg	vehicle + CUMS	i.g	8w	DGHKMN
Yuechen Guan	2021	China	Kunming mice	18–22 g	male	10/10	CUMS	quercetin + CUMS	40 mg/kg	double distilled water + CUMS	i.g	21 d	ABDKLN
Zhong-Xuan Ma	2021	China	ICR mice	22–24 g	male	3/3	CUMS	quercetin + CUMS	30 mg/kg	CUMS	i.g	3w	BCDI
Syeda Madiha	2021	Pakistan	Wistar rat	150–200 g	male	8/8	—	quercetin	50 mg/kg	blank control	p.o	14 d	DKLMN
Guoli Wang	2021	China	C57BL/6J mice	—	female	6/6	ERα-KO	quercetin + ERα-KO	100 mg/kg	vehicle + ERα-KO	p.o	10w	ACI
Diogo Ferreira Bicca	2021	Brazil	Swiss mice	—	male	7/7	Glyphosate-based herbicide (GBH)	quercetin + GBH	30 mg/kg	vehicle + GBH	i.g	30 d	ADEK
Zihu Tan	2022	China	C57BL/6 mice	24–30 g	male	6/6	Bilateral carotid artery stenosis (BCAS)/chronic restraint stress (CRS)	quercetin + BCAS/CRS	60 mg/kg	vehicle + BCAS/CRS	i.p	14 d	ABCDEGH
Princewill Ikechukwu Ugwu	2022	Nigeria	Swiss albino mice	25–30 g	male	7/7	Social defeat stress (SDS)	quercetin + SDS	100 mg/kg	vehicle + SDS	i.p	14 d	CFGIJKLMN
Yanrong Yang	2022	China	SD rat	180–200 g	male	10/10	Middle cerebral artery embolization (MCAO)-induced post-stroke depression (PSD)	quercetin + exercise + PSD	50 μg/kg	exercise + PSD	p.o	12w	CDGHI
Ramya Balasubramanian	2023	India	C57BL/6J mice	—	male	6/6	Repeated mild traumatic brain injury (rmTBI)	quercetin + rmTBI	50 mg/kg	rmTBI	p.o	7 d	A
Olusegun Adebayo Adeoluwa	2023	Nigeria	Rat	—	male	6/6	LPS	quercetin + LPS	50 mg/kg	vehicle + LPS	p.o	7 d	FG
Chenjie Ge	2023	China	C57BL/6J mice	18–20 g	male	6/6	Corticosterone (CORT)	quercetin + CORT	80 mg/kg	saline + CORT	i.g	2w	ABEFGHIKM
Siqi Jia	2023	China	SD rat	190–220 g	male	16/16	CUMS	quercetin + CUMS	50 mg/kg	vehicle + CUMS	i.g	8w	KLN
Olusegun Adebayo Adeoluwa	2024	Nigeria	Swiss mice	20–25 g	male	5/5	—	quercetin	100 mg/kg	vehicle	p.o	60min	AC
Mehedi Hasan Bappi	2024	Bangladesh	Swiss Albino mice	24–28 g	male	6/6	—	quercetin	50 mg/kg	vehicle	p.o	0.5 h	A
Dan Wang-1	2024	China	Wistar rat	190–230 g	female	12/12	Ovariectomy combined with chronic unpredictable mild stress (OVX-CUMS)	quercetin + OVX-CUMS	50 mg/kg	vehicle + OVX-CUMS	i.g	4w	DKLMN
Dan Wang-2	2024	China	Wistar rat	190–230 g	female	9/9	Perimenopausal depression	quercetin + Perimenopausal depression	50 mg/kg	vehicle + Perimenopausal depression	i.g	4w	ABEK
Longfei Du	2024	China	C57BL/6J mice	25–30 g	male	6/6	CUS	quercetin + CUS	75 mg/kg	vehicle + CUS	i.p	2w	ACDFGH
Chenjie Ge	2024	China	C57BL/6 mice	18–22 g	male	6/6	CUMS	quercetin + CUMS	50 mg/kg	saline + CUMS	i.g	4w	ACD
Mikhil Santosh Kore	2024	India	Swiss albino mice	20–25 g	male	8/8	CUMS	quercetin + CUMS	20 mg/kg	CUMS	p.o	3w	ACEFGHIJ
Bozhi Li	2024	China	Wistar rat	180–220 g	male	12/12	CUMS	quercetin + CUMS	50 mg/kg	pure water + CUMS	i.g	8w	ADE
YUANYUAN LI	2024	China	SD rat	180–220 g	male	10/10	CUMS	quercetin + CUMS	52.08 mg/kg	saline + CUMS	i.g	6w	BDFGH
Sajjad Makhdoomi	2024	Iran	BALB/c mice	20 ± 5 g	male	6/6	—	quercetin	25 mg/kg	blank control	p.o	21 d	ABCELMN
Qing Zhu	2024	China	BALB/c mice	—	female	5/5	4T1 cells and CORT to create a BCRD model	quercetin + BCRD	8 mg/kg	BCRD	i.g	25 d	ACD
Pan Su	2024	China	ICR mice	20 ± 2 g	male	10/10	LPS	quercetin + LPS	50 mg/kg	vehicle + LPS	i.g	14 d	BCD
Fatemeh Tavakol	2024	Iran	NMRI mice	10–12 g	male	6/6	Social isolation stress (SIS)	quercetin + SIS	40 mg/kg	saline + SIS	i.p	45min	AB
Mingyan Wang	2024	China	SD rat	140–160 g	male	3/3	CUMS	quercetin + CUMS	60 mg/kg	CUMS	i.g	4w	ABCDIJ
Yali Hou	2025	China	Wistar rat	190 ± 20 g	female	12/12	Ovariectomy (OVX) combined with CUMS	quercetin + OVX-CUMS	50 mg/kg	vehicle + OVX-CUMS	i.g	4w	GHKL

Abbreviations: Ctrl, control; QE, quercetin; SD, Sprague–Dawley; min, minute; d, days; w, weeks; i.g., intragastric injection; i.p., intraperitoneal injection; p.o., oral administration; CUMS, chronic unpredictable mild stress; CUS, chronic unpredicted stress; LPS, lipopolysaccharide.

Notes: A, FST; B, OFT; C, TST; D, SPT; E, EPM; F, IL-6, levels; G, TNF-α, levels; H, IL-1β levels; I, BDNF, levels; J, CORT, levels; K, GSH, levels; L, MDA, levels; M, CAT, levels; and N, SOD, levels.

**TABLE 3:** Quality assessment of included studies.

Author	Year	(1)	(2)	(3)	(4)	(5)	(6)	(7)	(8)	(9)	(10)
Muragundla Anjaneyulu	2003	Unclear risk	Low risk	High risk	Unclear risk	High risk	Low risk	High risk	Low risk	Low risk	Low risk
Sangeeta Pilkhwal Sah	2011	Low risk	Low risk	Unclear risk	Unclear risk	High risk	Low risk	Unclear risk	Low risk	Low risk	Low risk
Jianxiang Liu	2013	Unclear risk	Low risk	High risk	Unclear risk	High risk	Low risk	High risk	Low risk	Low risk	Low risk
Puneet Rinwa	2013	Low risk	Low risk	Unclear risk	High risk	High risk	Low risk	Unclear risk	Low risk	Low risk	Low risk
Dilpesh Jain	2014	Unclear risk	Low risk	High risk	Unclear risk	High risk	Low risk	High risk	Low risk	Low risk	Low risk
Sameha Merzoug	2014	Low risk	Low risk	High risk	Low risk	High risk	Low risk	High risk	Low risk	Low risk	Low risk
Iandra Holzmann	2015	Low risk	Low risk	Unclear risk	Low risk	High risk	Low risk	High risk	Low risk	Low risk	Low risk
Vineet Mehta	2017	Low risk	Low risk	Unclear risk	High risk	High risk	Low risk	High risk	Low risk	Low risk	Low risk
Redouane Rebai	2017	Low risk	Low risk	Unclear risk	Unclear risk	High risk	Low risk	High risk	Low risk	Low risk	Unclear risk
Tanveer Singh	2017	Low risk	Low risk	Unclear risk	Unclear risk	High risk	Low risk	High risk	Low risk	Low risk	Unclear risk
Mustajab Quraishi	2018	Low risk	Low risk	High risk	Low risk	High risk	Low risk	High risk	Low risk	Low risk	Unclear risk
Noreen Samad	2018	Low risk	Low risk	High risk	Low risk	High risk	Low risk	High risk	Low risk	Low risk	Unclear risk
Khadeeja Khan	2019	Low risk	Low risk	High risk	Low risk	High risk	Low risk	High risk	Low risk	Low risk	Low risk
Putri Anggreini	2019	Unclear risk	Low risk	High risk	Low risk	High risk	Low risk	High risk	Low risk	Low risk	Low risk
SRIRAM BS	2019	Unclear risk	Low risk	High risk	Unclear risk	High risk	Low risk	High risk	Low risk	Low risk	Low risk
Mohamed Lamine Toumi	2019	Unclear risk	Low risk	High risk	Unclear risk	High risk	Low risk	Unclear risk	Low risk	Low risk	Unclear risk
Fang Ke	2019	Low risk	Low risk	Unclear risk	Low risk	High risk	Low risk	Low risk	Low risk	Low risk	Low risk
Fella Boudiaf	2020	Unclear risk	Low risk	High risk	Unclear risk	High risk	Low risk	Unclear risk	Low risk	Low risk	Unclear risk
Francisco Donoso	2020	Low risk	Low risk	High risk	Unclear risk	High risk	Low risk	Low risk	Low risk	Low risk	Low risk
Jiajia Zhang	2020	Low risk	Low risk	Unclear risk	Low risk	High risk	Low risk	Low risk	Low risk	Low risk	Low risk
Shirin Sadighparvar	2020	Unclear risk	Low risk	High risk	Unclear risk	High risk	Low risk	Low risk	Low risk	Low risk	Low risk
Tuğçe DemirtaşŞahin	2020	Low risk	Low risk	Unclear risk	Low risk	High risk	Low risk	High risk	Low risk	Low risk	Low risk
Ismaeel Bin-Jaliah	2021	Low risk	Low risk	Unclear risk	Low risk	High risk	Low risk	High risk	Low risk	Low risk	Low risk
Anthony Taghogho Eduvière	2021	Unclear risk	Low risk	High risk	Unclear risk	High risk	Low risk	High risk	Low risk	Low risk	Low risk
Tong Guan	2021	Low risk	Low risk	Unclear risk	Low risk	High risk	Low risk	High risk	Low risk	Low risk	Low risk
Yuechen Guan	2021	Low risk	Low risk	Unclear risk	Low risk	High risk	Low risk	High risk	Low risk	Low risk	Low risk
Zhong-Xuan Ma	2021	Low risk	Low risk	Unclear risk	Low risk	High risk	Low risk	High risk	Low risk	Low risk	Low risk
Syeda Madiha	2021	Low risk	Low risk	Unclear risk	Low risk	High risk	Low risk	High risk	Low risk	Low risk	Low risk
Guoli Wang	2021	Low risk	Low risk	Unclear risk	Low risk	High risk	Low risk	High risk	Low risk	Low risk	Low risk
Diogo Ferreira Bicca	2021	Low risk	Low risk	Unclear risk	Low risk	High risk	Low risk	High risk	Low risk	Low risk	Low risk
Zihu Tan	2022	Low risk	Low risk	High risk	Low risk	High risk	Low risk	Low risk	Low risk	Low risk	Low risk
Princewill Ikechukwu Ugwu	2022	Low risk	Low risk	Unclear risk	High risk	High risk	Low risk	High risk	Low risk	Low risk	High risk
Yanrong Yang	2022	Low risk	Low risk	Unclear risk	Low risk	High risk	Low risk	High risk	Low risk	Low risk	Low risk
Ramya Balasubramanian	2023	Low risk	Low risk	Unclear risk	Low risk	High risk	Low risk	High risk	Low risk	Low risk	Low risk
Olusegun Adebayo Adeoluwa	2023	Low risk	Low risk	Unclear risk	Low risk	High risk	Low risk	High risk	Low risk	Low risk	Low risk
Chenjie Ge	2023	Low risk	Low risk	Unclear risk	Low risk	High risk	Low risk	High risk	Low risk	Low risk	Low risk
Siqi Jia	2023	Low risk	Low risk	Unclear risk	Low risk	High risk	Low risk	High risk	Low risk	Low risk	Low risk
Olusegun Adebayo Adeoluwa	2024	Unclear risk	Low risk	High risk	Unclear risk	High risk	Low risk	High risk	Low risk	Low risk	Unclear risk
Mehedi Hasan Bappi	2024	Unclear risk	Low risk	High risk	Unclear risk	High risk	Low risk	High risk	Low risk	Low risk	Unclear risk
Dan Wang-1	2024	Low risk	Low risk	High risk	Unclear risk	High risk	Low risk	Unclear risk	Low risk	Low risk	Low risk
Dan Wang-2	2024	Low risk	Low risk	High risk	Unclear risk	High risk	Low risk	Unclear risk	Low risk	Low risk	Low risk
Longfei Du	2024	Low risk	Low risk	Unclear risk	Low risk	High risk	Low risk	Low risk	Low risk	Low risk	Low risk
Chenjie Ge	2024	Low risk	Low risk	High risk	Low risk	High risk	Low risk	High risk	Low risk	Low risk	Unclear risk
Mikhil Santosh Kore	2024	Low risk	Low risk	Unclear risk	Low risk	High risk	Low risk	High risk	Low risk	Low risk	Low risk
Bozhi Li	2024	Low risk	Low risk	Unclear risk	Low risk	High risk	Low risk	High risk	Low risk	Low risk	Low risk
YUANYUAN LI	2024	Low risk	Low risk	Unclear risk	Low risk	High risk	Low risk	High risk	Low risk	Low risk	Unclear risk
Sajjad Makhdoomi	2024	Low risk	Low risk	Unclear risk	Low risk	High risk	Low risk	High risk	Low risk	Low risk	Unclear risk
Qing Zhu	2024	Low risk	Low risk	Unclear risk	Low risk	High risk	Low risk	High risk	Low risk	Low risk	Unclear risk
Pan Su	2024	Low risk	Low risk	Unclear risk	Low risk	High risk	Low risk	High risk	Low risk	Low risk	Low risk
Fatemeh Tavakol	2024	Low risk	Low risk	Unclear risk	Low risk	High risk	Low risk	Low risk	Low risk	Low risk	Low risk
Mingyan Wang	2024	Low risk	Low risk	Unclear risk	Unclear risk	High risk	Low risk	Unclear risk	Low risk	Low risk	Low risk
Yali Hou	2025	Low risk	Low risk	High risk	Low risk	High risk	Low risk	High risk	Low risk	Low risk	Unclear risk

Notes: (1) Random sequence generation; (2) Baseline characteristics; (3) Allocation concealment; (4) Random housing; (5) Blinding (for animal breeders and researchers); (6) Random outcome assessment; (7) Blinding (for outcome evaluators); (8) Incomplete outcome data; (9) Selective outcome reporting; (10) Other risk of bias.

### 3.3 Behavioral tests

#### 3.3.1 FST

**FIGURE 2:**
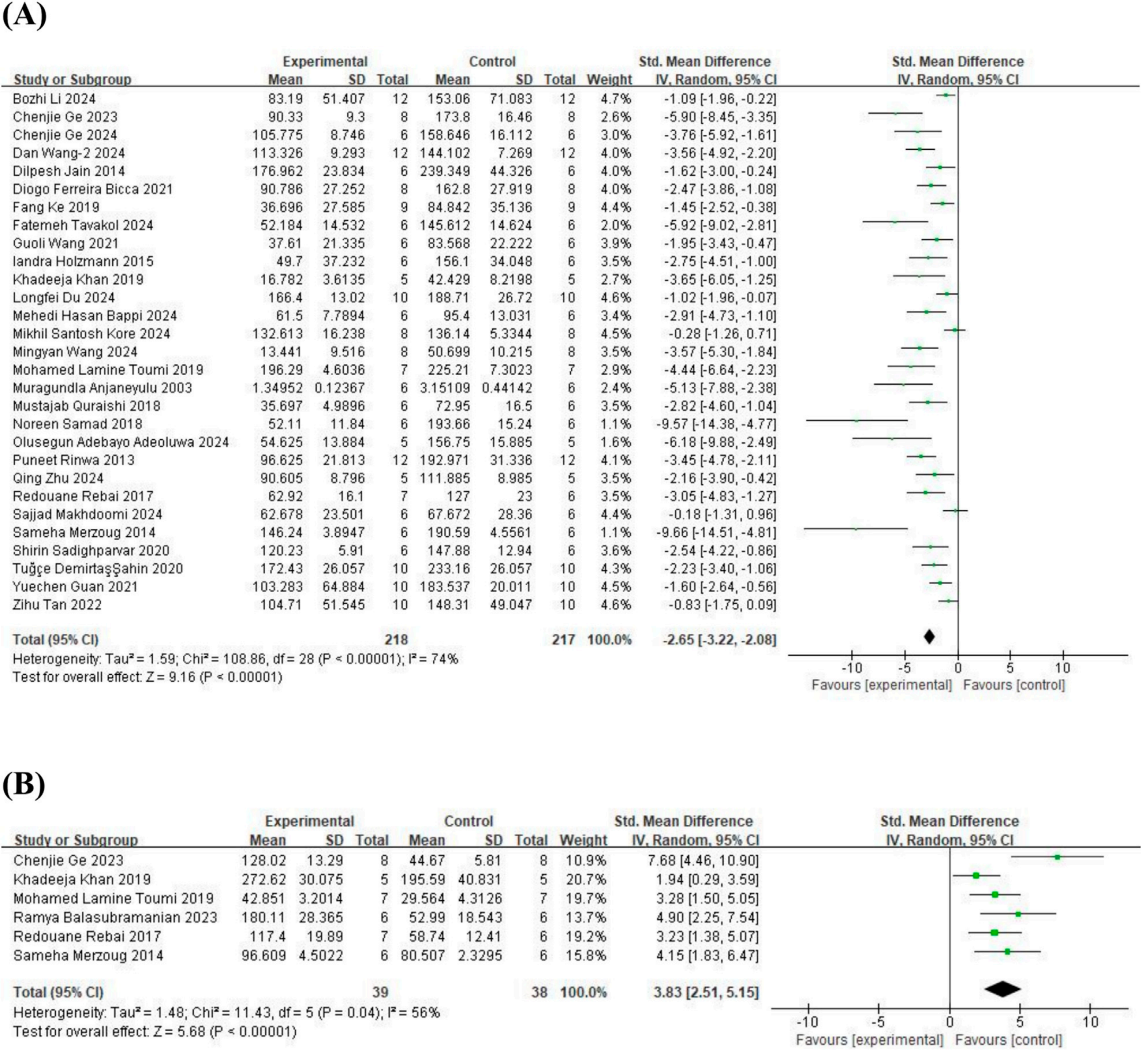
Forest plot for the effect of quercetin on the forced swimming test. **(A)** the immobility time; **(B)** the swimming time.

The meta-analysis of FST, 29 studies (Anjaneyulu et al., 2003; Rinwa and Kumar, 2013; Jain and Gangshettiwar, 2014; Merzoug et al., 2014; Holzmann et al., 2015; Rebai et al., 2017; Quraishi et al., 2018; Samad et al., 2018; Fang et al., 2019; Khan et al., 2019; Toumi et al., 2019; Ahin et al., 2020; Sadighparvar et al., 2020; Bicca et al., 2021; Guan T. et al., 2021; Wang et al., 2021; Tan et al., 2022; Ge et al., 2023; Adeoluwa et al., 2024; Bappi et al., 2024; Du et al., 2024; Ge et al., 2024; Kore et al., 2024; Li B. et al., 2024; Makhdoomi et al., 2024; Tavakol et al., 2024; Wang et al., 2024c; Wang et al., 2024b; Zhu et al., 2024) on immobility time (involving 435 animals) and 6 studies (Merzoug et al., 2014; Rebai et al., 2017; Khan et al., 2019; Toumi et al., 2019; Balasubramanian et al., 2023; Ge et al., 2023) on swimming time (involving 77 animals) were included. The results indicated that, compared to the control group, quercetin treatment significantly reduced immobility time (SMD = −2.65; 95% CI = [−3.22, −2.08]; p < 0.001; *I* ^*2*^ = 74%) and increased swimming time (SMD = 3.83; 95% CI = [2.51, 5.15]; p < 0.001; *I* ^*2*^ = 56%). The forest plot showing the effect of quercetin on FST is presented in Figure 2.

#### 3.3.2 OFT

**FIGURE 3:**
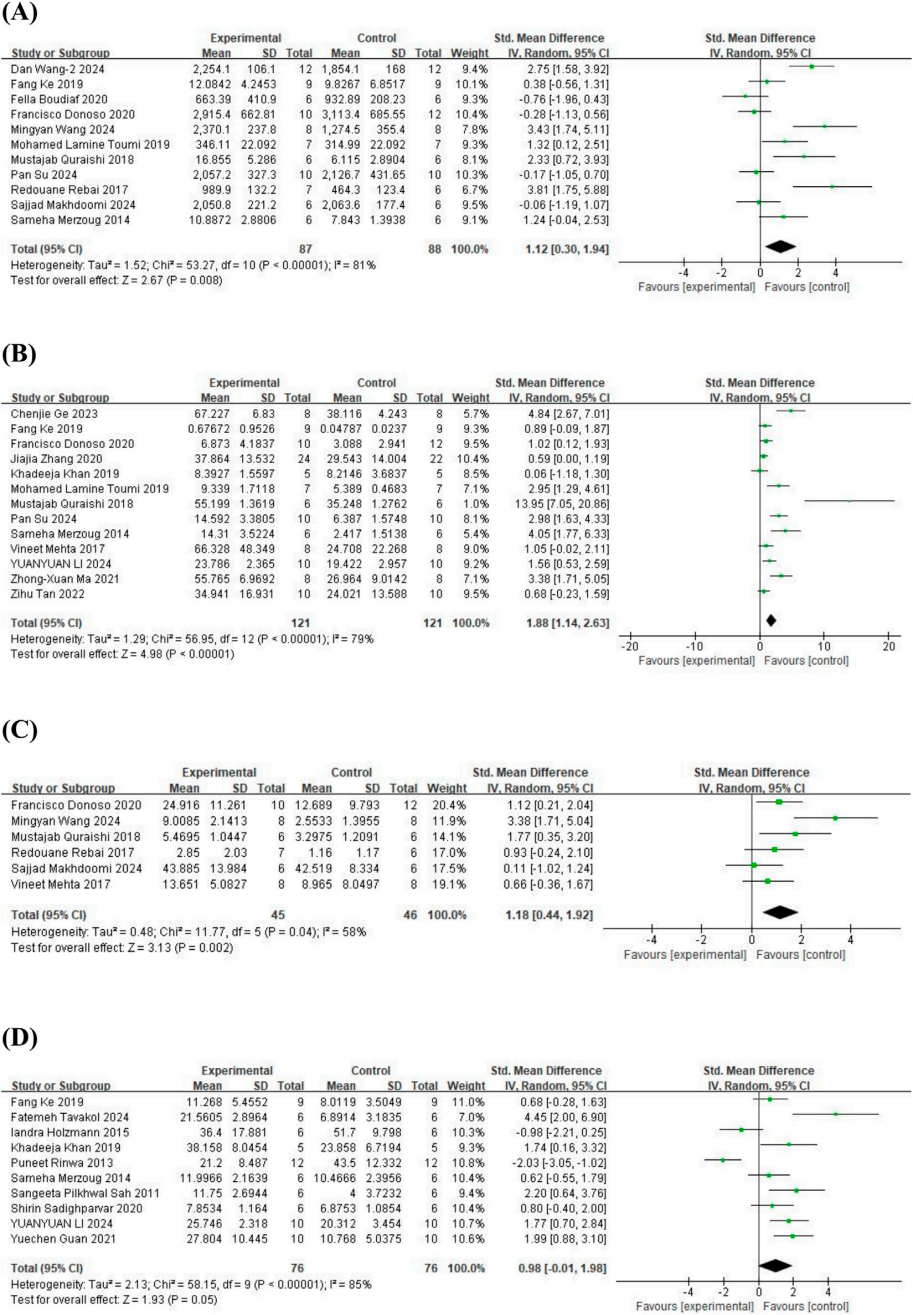
Forest plot for the effect of quercetin on the open field test. **(A)** Total distance traveled; **(B)** the time spent in the central area; **(C)** the number of entries into the central; **(D)** the number of standing episodes.

As for the OFT, 11 studies (Merzoug et al., 2014; Rebai et al., 2017; Quraishi et al., 2018; Fang et al., 2019; Toumi et al., 2019; Boudia et al., 2020; Donoso et al., 2020; Makhdoomi et al., 2024; Su et al., 2024; Wang et al., 2024c; Wang et al., 2024b) on total distance traveled (involving 175 animals), 13 studies (Merzoug et al., 2014; Mehta et al., 2017; Quraishi et al., 2018; Fang et al., 2019; Khan et al., 2019; Toumi et al., 2019; Donoso et al., 2020; Zhang et al., 2020; Ma et al., 2021; Tan et al., 2022; Ge et al., 2023; Li B. et al., 2024; Su et al., 2024) on time spent in the central area (involving 242 animals), 6 studies (Mehta et al., 2017; Rebai et al., 2017; Quraishi et al., 2018; Donoso et al., 2020; Makhdoomi et al., 2024; Wang et al., 2024c) on the number of entries into the central area (involving 91 animals), and 10 studies (Sah et al., 2011; Rinwa and Kumar, 2013; Merzoug et al., 2014; Holzmann et al., 2015; Fang et al., 2019; Khan et al., 2019; Sadighparvar et al., 2020; Guan T. et al., 2021; Li B. et al., 2024; Tavakol et al., 2024) on the number of standing episodes (involving 152 animals) were included. The results showed that, compared to the control group, quercetin treatment significantly increased the total distance traveled (SMD = 1.12; 95% CI = [0.30, 1.94]; p = 0.008; *I* ^*2*^ = 81%), time spent in the central area (SMD = 1.88; 95% CI = [1.14, 2.63]; p < 0.001; *I* ^*2*^ = 79%), and the number of entries into the central area (SMD = 1.18; 95% CI = [0.44, 1.92]; p = 0.002; *I* ^*2*^ = 58%). However, quercetin treatment did not show a statistically significant effect on the number of standing episodes (SMD = 0.98; 95% CI = [−0.01, 1.98]; p = 0.05; *I* ^*2*^ = 85%). The forest plot showing the effect of quercetin on OFT is presented in Figure 3.

#### 3.3.3 TST

**FIGURE 4:**
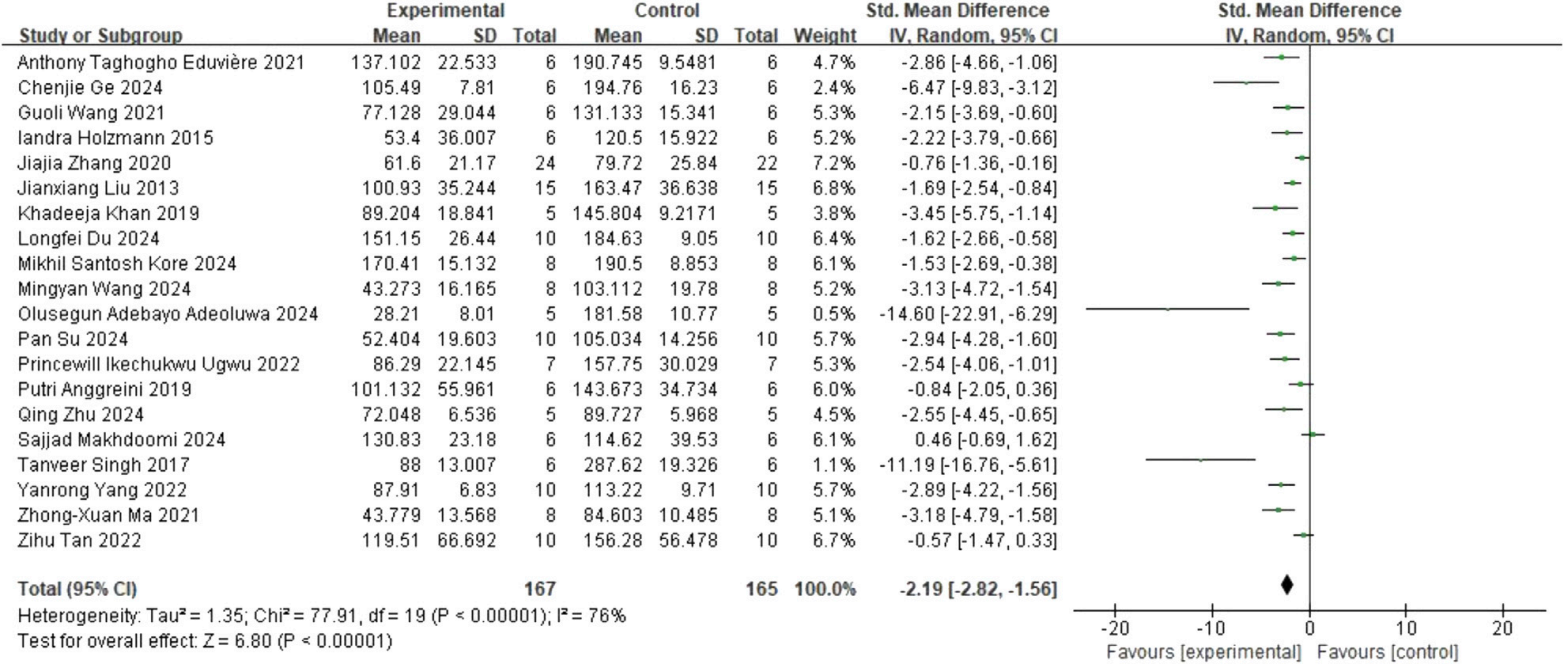
Forest plot for the effect of quercetin on the tail suspension test.

In the TST, 20 studies (Liu et al., 2013; Holzmann et al., 2015; Singh et al., 2017; Anggreini et al., 2019; Khan et al., 2019; Zhang et al., 2020; Eduviere et al., 2021; Ma et al., 2021; Wang et al., 2021; Tan et al., 2022; Ugwu et al., 2022; Yang et al., 2022; Adeoluwa et al., 2024; Du et al., 2024; Ge et al., 2024; Kore et al., 2024; Makhdoomi et al., 2024; Su et al., 2024; Wang et al., 2024c; Zhu et al., 2024) on immobility time (involving 332 animals) were included. The results indicated that, compared to the control group, quercetin treatment significantly reduced immobility time (SMD = −2.19; 95% CI = [-2.82, −1.56]; p < 0.001; *I* ^*2*^ = 76%). The forest plot showing the effect of quercetin on TST is presented in Figure 4.

#### 3.3.4 SPT

**FIGURE 5:**
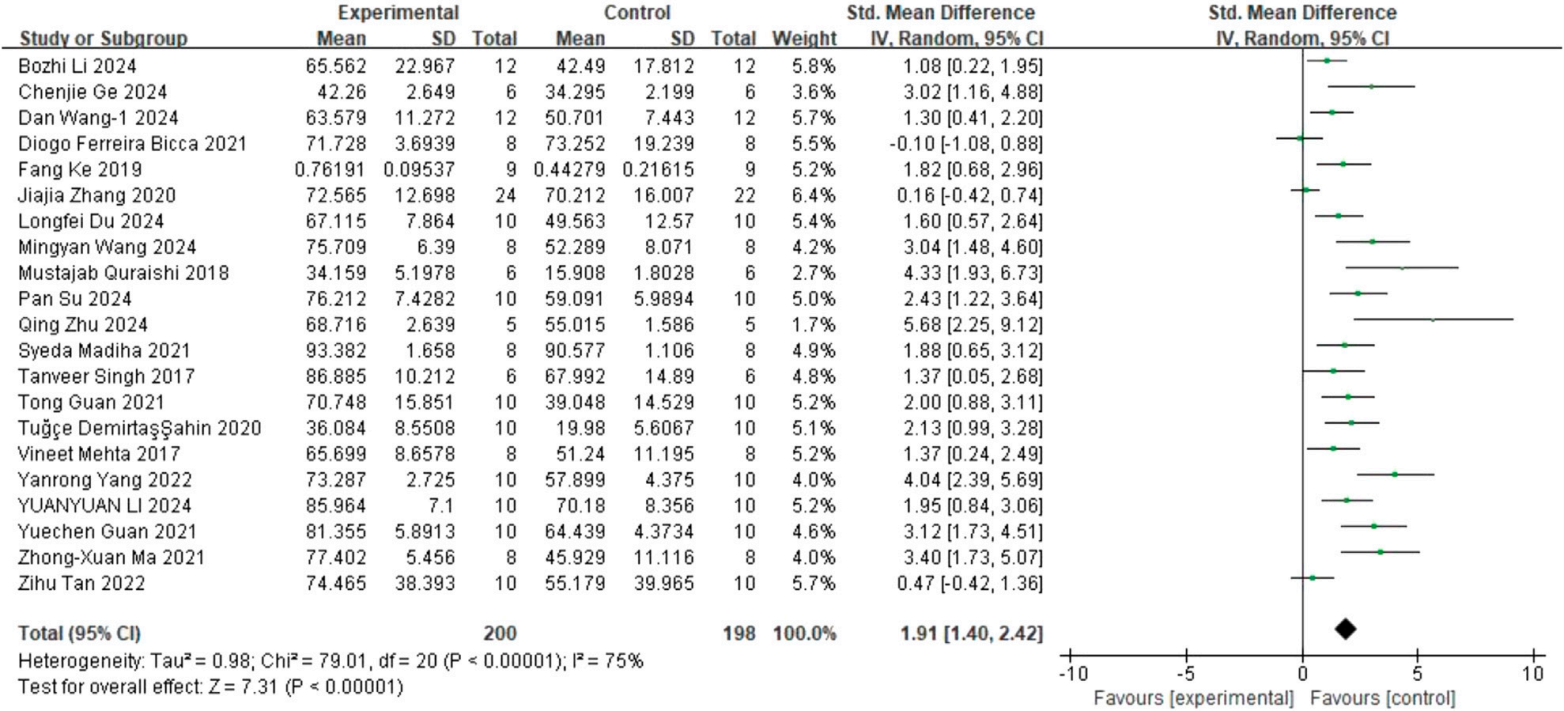
Forest plot for the effect of quercetin on the sucrose preference test.

Regarding SPT, 21 studies (Mehta et al., 2017; Singh et al., 2017; Quraishi et al., 2018; Fang et al., 2019; Ahin et al., 2020; Zhang et al., 2020; Bicca et al., 2021; Guan T. et al., 2021; Guan Y. et al., 2021; Ma et al., 2021; Madiha et al., 2021; Tan et al., 2022; Yang et al., 2022; Du et al., 2024; Ge et al., 2024; Li B. et al., 2024; Li B. et al., 2024; Su et al., 2024; Wang et al., 2024c; Wang et al., 2024a; Zhu et al., 2024) on sucrose preference (involving 398 animals) were included. The results indicated that, compared to the control group, quercetin treatment significantly increased sucrose preference in animals (SMD = 1.91; 95% CI = [1.40, 2.42]; p < 0.001; *I* ^*2*^ = 75%). The forest plot showing the effect of quercetin on SPT is presented in Figure 5.

#### 3.3.5 EPM

**FIGURE 6:**
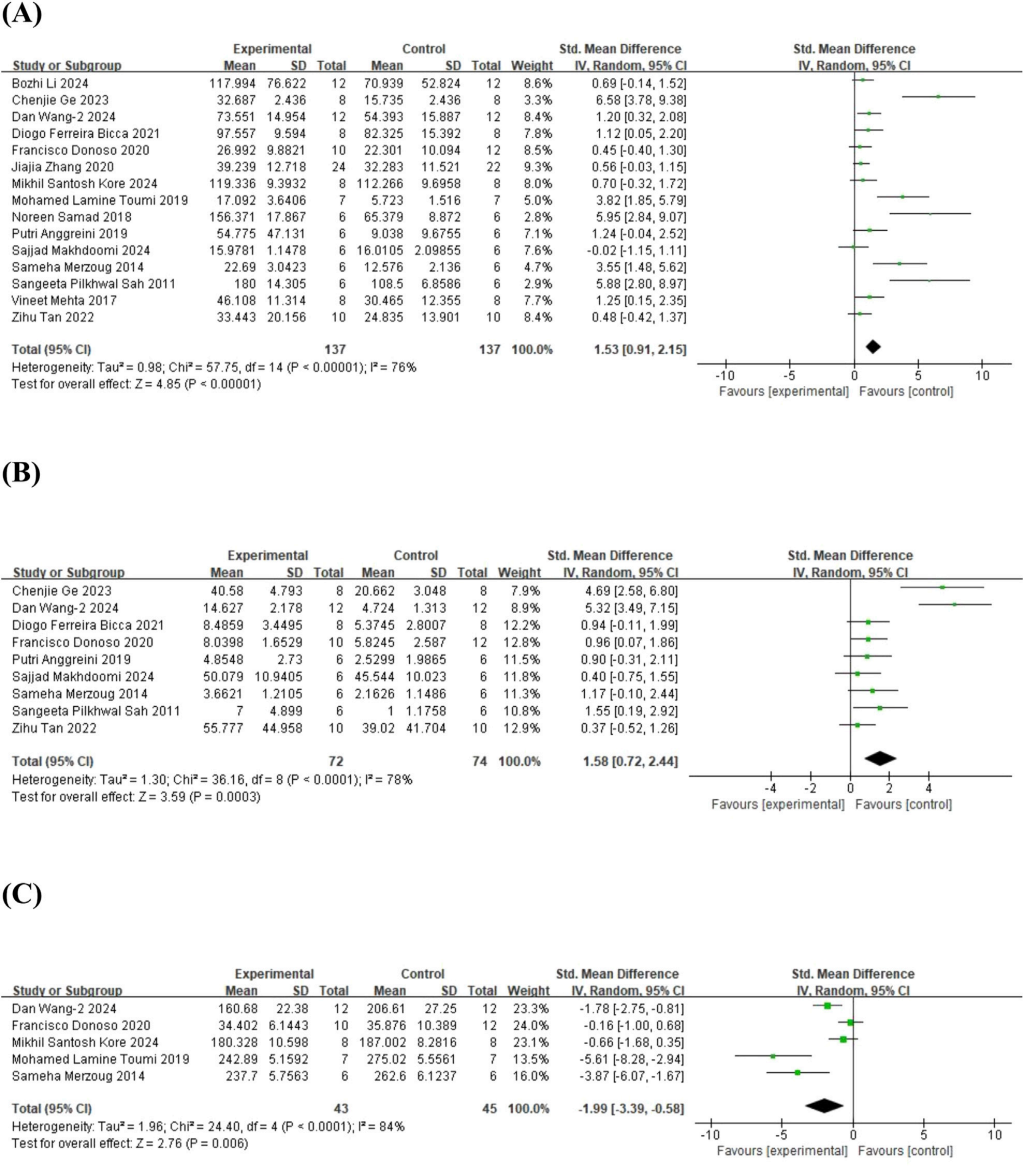
Forest plot for the effect of quercetin on the elevated plus maze. **(A)** The time spent in the open arms; **(B)** the number of entries into the open arms; **(C)** the time spent closed arms.

In terms of the EPM, 15 studies (Sah et al., 2011; Merzoug et al., 2014; Mehta et al., 2017; Samad et al., 2018; Anggreini et al., 2019; Toumi et al., 2019; Donoso et al., 2020; Zhang et al., 2020; Bicca et al., 2021; Tan et al., 2022; Ge et al., 2023; Kore et al., 2024; Li B. et al., 2024; Makhdoomi et al., 2024; Wang et al., 2024b) on time spent in the open arms (involving 274 animals), 9 studies (Sah et al., 2011; Merzoug et al., 2014; Anggreini et al., 2019; Donoso et al., 2020; Bicca et al., 2021; Tan et al., 2022; Ge et al., 2023; Makhdoomi et al., 2024; Wang et al., 2024b) on the number of entries into the open arms (involving 146 animals), and 5 studies (Merzoug et al., 2014; Toumi et al., 2019; Donoso et al., 2020; Kore et al., 2024; Wang et al., 2024b) on time spent in the closed arms (involving 88 animals) were included. The results showed that, compared to the control group, quercetin treatment significantly increased the time spent in the open arms (SMD = 1.53; 95% CI = [0.91, 2.15]; p < 0.001; *I* ^*2*^ = 76%), the number of entries into the open arms (SMD = 1.58; 95% CI = [0.72, 2.44]; p < 0.001; *I* ^*2*^ = 78%), and decreased the time spent in the closed arms (SMD = −1.99; 95% CI = [-3.39, −0.58]; p = 0.006; *I* ^*2*^ = 84%). The forest plot showing the effect of quercetin on EPM is presented in Figure 6.

### 3.4 Biochemical assay

#### 3.4.1 Oxidative stress

**FIGURE 7:**
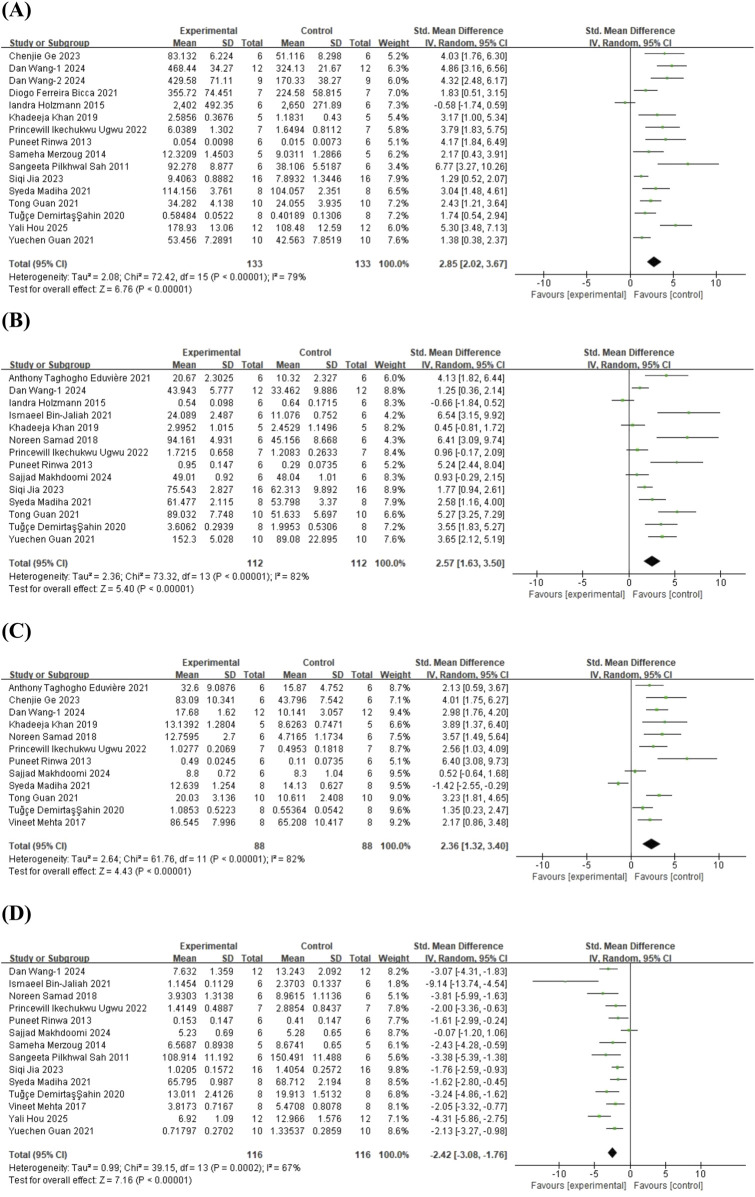
Forest plot for the effect of quercetin on the antioxident effects. **(A)** GSH; **(B)** SOD; **(C)** CAT; **(D)** MDA.

To investigate the antioxidant effects of quercetin treatment, 16 studies (Sah et al., 2011; Rinwa and Kumar, 2013; Merzoug et al., 2014; Holzmann et al., 2015; Khan et al., 2019; Ahin et al., 2020; Bicca et al., 2021; Guan T. et al., 2021; Guan Y. et al., 2021; Madiha et al., 2021; Ugwu et al., 2022; Ge et al., 2023; Jia et al., 2023; Wang et al., 2024a; Wang et al., 2024b; Hou et al., 2025) on glutathione (GSH) (involving 266 animals), 14 studies (Rinwa and Kumar, 2013; Holzmann et al., 2015; Samad et al., 2018; Khan et al., 2019; Ahin et al., 2020; Bin-Jaliah, 2021; Eduviere et al., 2021; Guan T. et al., 2021; Guan Y. et al., 2021; Madiha et al., 2021; Ugwu et al., 2022; Jia et al., 2023; Makhdoomi et al., 2024; Wang et al., 2024a) on superoxide dismutase (SOD) (involving 224 animals), 12 studies (Rinwa and Kumar, 2013; Mehta et al., 2017; Samad et al., 2018; Khan et al., 2019; Ahin et al., 2020; Eduviere et al., 2021; Guan T. et al., 2021; Madiha et al., 2021; Ugwu et al., 2022; Ge et al., 2023; Makhdoomi et al., 2024; Wang et al., 2024a) on catalase (CAT) (involving 176 animals), and 14 studies (Sah et al., 2011; Rinwa and Kumar, 2013; Merzoug et al., 2014; Mehta et al., 2017; Samad et al., 2018; Ahin et al., 2020; Bin-Jaliah, 2021; Guan T. et al., 2021; Madiha et al., 2021; Ugwu et al., 2022; Jia et al., 2023; Makhdoomi et al., 2024; Wang et al., 2024a; Hou et al., 2025) on malondialdehyde (MDA) (involving 232 animals) were included. The results indicated that, compared to the control group, quercetin treatment significantly increased GSH levels in animals (SMD = 2.85; 95% CI = [2.02, 3.67]; p < 0.001; *I* ^*2*^ = 79%), SOD levels (SMD = 2.57; 95% CI = [1.63, 3.50]; p < 0.001; *I* ^*2*^ = 82%), and CAT levels (SMD = 2.36; 95% CI = [1.32, 3.40]; p < 0.001; *I* ^*2*^ = 82%), while decreasing MDA levels (SMD = −2.42; 95% CI = [-3.08, −1.76]; p < 0.001; *I* ^*2*^ = 67%). The forest plot showing the effect of quercetin on oxidative stress markers is presented in Figure 7.

#### 3.4.2 Inflammatory cytokines

**FIGURE 8:**
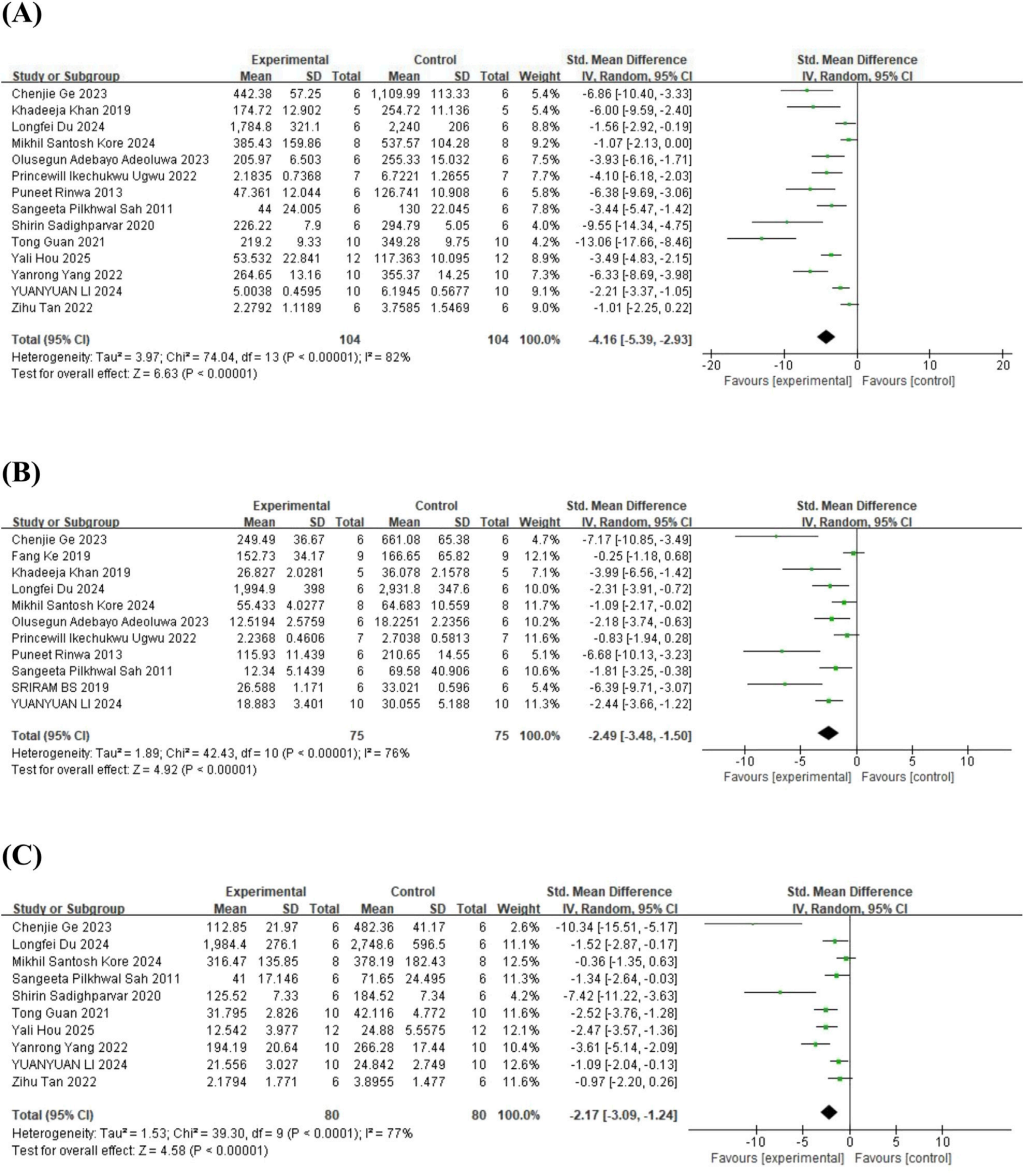
Forest plot for the effect of quercetin on the inflammatory cytokines. **(A)** TNF-α; **(B)** IL-6; **(C)** IL-1β.

In the case of inflammatory cytokines, 14 studies (Sah et al., 2011; Rinwa and Kumar, 2013; Khan et al., 2019; Sadighparvar et al., 2020; Guan T. et al., 2021; Tan et al., 2022; Ugwu et al., 2022; Yang et al., 2022; Adeoluwa et al., 2023; Ge et al., 2023; Du et al., 2024; Kore et al., 2024; Li B. et al., 2024; Hou et al., 2025) on tumor necrosis factor-α (TNF-α) (involving 208 animals), 11 studies (Sah et al., 2011; Rinwa and Kumar, 2013; Fang et al., 2019; Khan et al., 2019; Sriram and Ravichandra, 2019; Ugwu et al., 2022; Adeoluwa et al., 2023; Ge et al., 2023; Du et al., 2024; Kore et al., 2024; Li B. et al., 2024) on interleukin-6 (IL-6) (involving 150 animals), and 10 studies (Sah et al., 2011; Sadighparvar et al., 2020; Guan T. et al., 2021; Tan et al., 2022; Yang et al., 2022; Ge et al., 2023; Du et al., 2024; Kore et al., 2024; Li B. et al., 2024; Hou et al., 2025) on interleukin-1β (IL-1β) (involving 160 animals) were included in the meta-analysis. The results indicated that, compared to the control group, quercetin treatment significantly reduced TNF-α levels in animals (SMD = −4.16; 95% CI = [-5.39, −2.93]; p < 0.001; *I* ^*2*^ = 82%), IL-6 levels (SMD = −2.49; 95% CI = [-3.48, −1.50]; p < 0.001; *I* ^*2*^ = 76%), and IL-1β levels (SMD = −2.17; 95% CI = [-3.09, −1.24]; p < 0.001; *I* ^*2*^ = 77%). These results suggest that quercetin has significant anti-inflammatory effects. The forest plot showing the effect of quercetin on inflammatory cytokine levels is presented in Figure 8.

#### 3.4.3 BDNF and CORT

**FIGURE 9:**
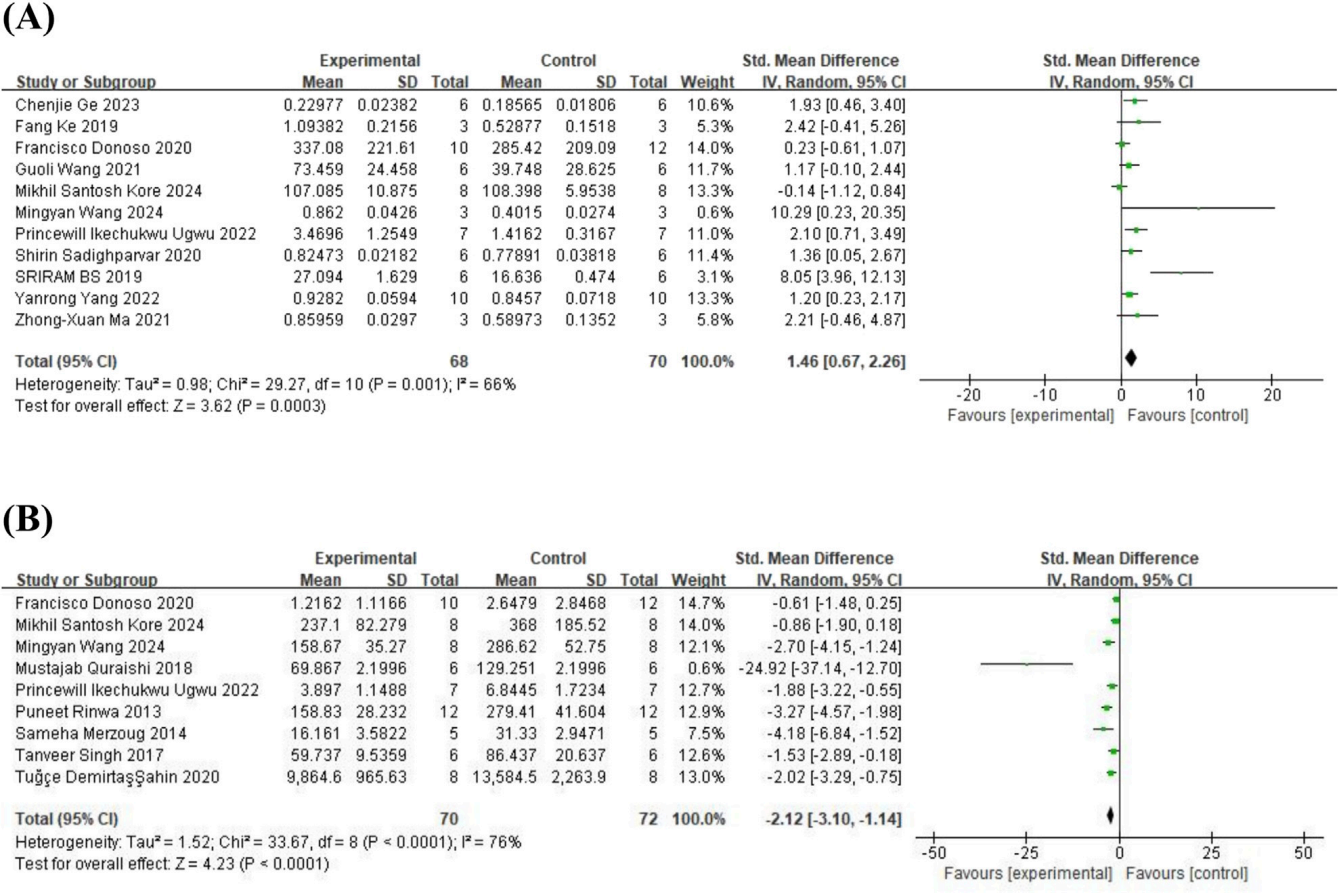
Forest plot for the effect of quercetin on BDNF and CORT levels. **(A)** BDNF; **(B)** CORT.

Eleven studies (Fang et al., 2019; Sriram and Ravichandra, 2019; Donoso et al., 2020; Sadighparvar et al., 2020; Ma et al., 2021; Wang et al., 2021; Ugwu et al., 2022; Yang et al., 2022; Ge et al., 2023; Kore et al., 2024; Wang et al., 2024c) on brain-derived neurotrophic factor (BDNF) (involving 138 animals) and 9 studies (Rinwa and Kumar, 2013; Merzoug et al., 2014; Singh et al., 2017; Quraishi et al., 2018; Donoso et al., 2020; Ahin et al., 2020; Ugwu et al., 2022; Kore et al., 2024; Wang et al., 2024c) on corticosterone (CORT) (involving 142 animals) were included. The results indicated that, compared to the control group, quercetin treatment significantly increased BDNF levels (SMD = 1.46; 95% CI = [0.67, 2.26]; p < 0.001; *I* ^*2*^ = 66%) and decreased CORT levels in animals (SMD = −2.12; 95% CI = [-3.10, −1.14]; p < 0.001; *I* ^*2*^ = 76%). The forest plot showing the effect of quercetin on BDNF and CORT levels is presented in Figure 9. The effects of quercetin on all behavioral and biochemical endpoints are summarized in Table 4.

**TABLE 4:** Summary of the effects of quercetin vs. control on all behavioral and biochemical endpoints.

Category	Outcome	Specific measure	NO. of studies	Heterogeneity		SMD	95%CI		*P* Value
				*I* ^*2*^ (%)	*P* Value				
Behavioral tests	FST	Immobility time	29	74	<0.001	−2.65	−3.22	−2.08	<0.001
		Swimming time	6	56	0.04	3.83	2.51	5.15	<0.001
	OFT	Total distance traveled	11	81	<0.001	1.12	0.30	1.94	0.008
		Time spent in central area	13	79	<0.001	1.88	1.14	2.63	<0.001
		Number of entries into central area	6	58	0.04	1.18	0.44	1.92	0.002
		Number of standing episodes	10	85	<0.001	0.98	−0.01	1.98	0.05
	TST	Immobility time	20	76	<0.001	−2.19	−2.82	−1.56	<0.001
	SPT	Sucrose preference	21	75	<0.001	1.91	1.40	2.42	<0.001
	EPM	Number of entries into open arms	9	78	<0.001	1.58	0.72	2.44	<0.001
		Time spent in open arms	15	76	<0.001	1.53	0.91	2.15	<0.001
		Time spent in closed arms	5	84	<0.001	−1.99	−3.39	−0.58	0.006
Biochemical assay	Oxidative stress	GSH	16	79	<0.001	2.85	2.02	3.67	<0.001
		MDA	14	67	<0.001	−2.42	−3.08	−1.76	<0.001
		CAT	12	82	<0.001	2.36	1.32	3.40	<0.001
		SOD	14	82	<0.001	2.57	1.63	3.50	<0.001
	Inflammatory cytokines	IL-6	11	76	<0.001	−2.49	−3.48	−1.50	<0.001
		TNF-α	14	82	<0.001	−4.16	−5.39	−2.93	<0.001
		IL-1β	10	77	<0.001	−2.17	−3.09	−1.24	<0.001
	Neurofactor	BDNF	11	66	0.001	1.46	0.67	2.26	<0.001
		CORT	9	76	<0.001	−2.12	−3.10	−1.14	<0.001

Abbreviations: CI, credibility interval; SMD, standardized mean difference; FST, forced swimming test; TST, tail suspension test; SPT, sucrose preference test; OFT, open field test; EPM, elevated plus maze; CORT, corticosterone; BDNF, brain-derived neurotrophic factor; CAT, catalase; MDA, malondialdehyde; SOD, superoxide dismutase; GSH, glutathione; IL-1β, interleukin-1β; IL-6, interleukin-6; TNF-α, tumor necrosis factor-α.

### 3.5 Sensitivity analysis and publication bias

In the sensitivity analysis with the exclusion of one study, the overall effects for all outcomes remained consistent, except for the number of standing episodes in the open field test. In the analysis of standing episodes, after excluding the study by Iandra Holzmann et al., the results changed. Heterogeneity decreased, and a significant effect was observed (SMD = 1.21; 95% CI = [0.17, 2.25]; p = 0.02; *I* ^*2*^ = 84%). After excluding the study by Puneet Rinwa et al., the results also changed. Heterogeneity decreased, and a significant effect was observed (SMD = 1.28; 95% CI = [0.51, 2.06]; p = 0.001; *I* ^*2*^ = 70%). The detailed results of the sensitivity analysis are presented in Supplementary Figure S1.

Funnel plots, Begg’s test, and Egger’s test were conducted for 15 outcome measures, including FST immobility time, total distance traveled in OFT, number of standing episodes in OFT, time spent in the central area of OFT, TST immobility time, sucrose preference in SPT, time spent in the open arms of the EPM, GSH, SOD, CAT, MDA, TNF-α, IL-6, IL-1β, and BDNF, to assess publication bias. The results indicated that there is some publication bias in the study (see Supplementary Figures S2-S3). Despite the positive findings, further research is needed to validate these results due to the potential publication bias in this meta-analysis.

### 3.6 Subgroup analysis

Due to the high heterogeneity among the meta-analyses, we further conducted subgroup analyses of all behavioral tests and biochemical markers according to animal species, dose of quercetin intervention, and duration of treatment. Subgroup analysis demonstrated that, compared to the control group, quercetin administration at a dose of ≥60 mg/kg significantly increased the total distance traveled in the open field test (OFT) (SMD = 1.88; 95% CI = [0.63, 3.12]; p = 0.003; *I* ^*2*^ = 59%). Conversely, no statistically significant differences were observed in the total distance traveled in the OFT at doses of ≤10 mg/kg or 10–60 mg/kg. The influence of shorter treatment durations (≤1 week) on total distance traveled was not significant (SMD = 0.97; 95% CI = [-0.29, 2.24]; p = 0.13; *I* ^*2*^ = 73%). In contrast, longer treatment durations (2–4 weeks) significantly increased the total distance traveled (SMD = 2.39; 95% CI = [0.52, 4.25]; p = 0.01; *I* ^*2*^ = 85%). In subgroup analyses by species, treatment with quercetin significantly increased the total distance traveled in rats (SMD = 1.44; 95% CI = [0.46, 2.43]; p = 0.004; *I* ^*2*^ = 83%), whereas no significant effect was observed in inbred mice (SMD = −0.06; 95% CI = [-1.19, 1.07]; p = 0.92). Detailed results of the subgroup analyses are shown in Supplementary Table S1.

## 4 Discussion

In this meta-analysis, we summarized the evidence from 52 published animal studies, which investigated the effects of quercetin supplementation on antidepressant effects across various behavioral and biochemical parameters. The results indicate that quercetin significantly affected animal behavior measures, including immobility time and swimming time in the FST, total distance traveled, time spent in the central area, and the number of entries into the central area in the OFT, immobility time in the TST, sucrose preference in the SPT, the number of entries into the open arms, time spent in the open arms, and time spent in the closed arms in the EPM, as well as biochemical markers such as GSH, SOD, CAT, MDA, TNF-α, IL-6, IL-1β, BDNF, and CORT. However, no significant effect was found on the number of standing episodes in the OFT.

This study shows that, compared to the control group, quercetin significantly increased sucrose preference in the sucrose preference test, reduced immobility time in the forced swimming test (FST) and tail suspension test (TST), and decreased time spent in the closed arms in the elevated plus maze (EPM). Additionally, quercetin increased total distance traveled and had significant effects on central area time and the number of entries into the open arms in the open field test (OFT) and EPM, showing the great effciency of quercetin for relieving depressive symptoms in animal studies. Depressive behavior test indicators are crucial for assessing the progression and therapeutic response of depression. Typically, the more severe the depression, the longer the immobility time, the shorter the distance traveled, and the less time spent in the central area and open arms, with lower sucrose preference (Tong et al., 2023; Ma et al., 2024). As reported by Anggreini, mice under stress showed a reduction in the time spent in open arms and the number of open-arm entries, indicating reduced exploratory activity, which is characteristic of higher anxiety in mice (Anggreini et al., 2019). Ma et al. found that chronic quercetin supplementation enhanced sucrose preference in mice subjected to chronic unpredictable mild stress, alleviating one of the key factors of depressive behavior—anhedonia (Ma et al., 2021). Furthermore, Samad et al. reported that quercetin can prevent stress-induced anxiety and depressive-like behaviors, as well as improve memory in male mice (Samad et al., 2018). In addition, Anjaneyu Lu et al.'s research on the antidepressant activity of quercetin in diabetic rats showed that quercetin reduced immobility time in a dose-dependent manner, which was similar to the effect of the antipsychotic drug fluoxetine and imipramine (Anjaneyulu et al., 2003). Moreover, the antidepressant and anxiolytic-like effects observed in quercetin-treated animals in our study are consistent with the findings of Sameha Merzoug, who reported that quercetin alleviates doxorubicin-induced anxiety-like behaviors and motor dysfunction in the open field and elevated plus maze tests, except for vertical exploration (i.e., the number of standing episodes) (Merzoug et al., 2014).

This study shows that quercetin significantly increases the levels of GSH, SOD, and CAT in animals, and decreases the levels of MDA, TNF-α, IL-6, and IL-1β. The anti-inflammatory and antioxidant effects of quercetin may be one of the important mechanisms underlying its antidepressant effects. Many studies suggest that the anti-inflammatory effects of quercetin may help alleviate neuropsychiatric symptoms. Sah et al. demonstrated that quercetin significantly reduced the levels of IL-1β and IL-6 in rats treated with lipopolysaccharide (LPS), thereby improving anxiety-like symptoms (Sah et al., 2011). These results were also verified in mice subjected to chronic unpredictable stress (CUS) (Mehta et al., 2017; Du et al., 2024). Due to the presence of both catechol and hydroxyl functional groups in its molecular structure, quercetin can directly exert antioxidant effects (Qi et al., 2022). Previously, Şahin et al. showed that quercetin, when administered via intraperitoneal injection, increased the SOD activity in the striatum of rats in the CUMS model (Ahin et al., 2020). One study found that quercetin had a significant effect on the perimenopausal depression rat model, as quercetin treatment significantly increased GSH levels in the brain and reduced the level of the oxidative stress marker MDA (Hou et al., 2025). Another study showed that quercetin alleviated oxidative stress and inflammation in rats by upregulating antioxidant mechanisms and downregulating the expression of COX2 and NF-κB (Bahar et al., 2017).

This study demonstrates that quercetin significantly increases BDNF levels and decreases CORT levels in animals, further supporting the biological basis of its antidepressant action. BDNF is a protein abundantly present in the human brain, which plays a critical role in protecting dendrites and axons, promoting synaptic plasticity, and regulating neuronal survival and intracellular signaling pathways (Kowiański et al., 2018). Clinical studies have shown that BDNF levels are significantly reduced in patients diagnosed with major depressive disorder (Kishi et al., 2017; Shi et al., 2020). CORT is an important glucocorticoid that regulates the body’s response to various stressors, such as psychological and physiological stress. A large body of evidence indicates that patients with depression or those who are chronically stressed typically exhibit overactivation of the hypothalamic-pituitary-adrenal (HPA) axis and elevated cortisol levels (Cubała and Landowski, 2014; Fischer et al., 2017; Druzhkova et al., 2022). Furthermore, existing studies have demonstrated that quercetin and its derivatives may exert neuroprotective effects by interacting with NMDA receptors, thereby reducing neuronal hyperexcitability and damage (Subramanian et al., 2023). Additionally, the modulation of NMDA receptors by quercetin could help restore neurotransmitter balance and ameliorate depressive-like behaviors (Wang et al., 2024c).

The findings of this study may provide multifaceted reference information for the future design of clinical trials of quercetin, which could facilitate the development of quercetin as a potential antidepressant and offer more therapeutic options for patients with depression. First, regarding the selection of subjects, the animal models included in this study cover a variety of methods for inducing depression, suggesting that future clinical trials may consider including patient groups with different etiologies or clinical manifestations of depression to more comprehensively evaluate the efficacy of quercetin. Second, in terms of dosing regimen design, this study has a wide range of quercetin doses and diverse routes of administration. Future clinical trials can refer to this information and, in combination with human pharmacokinetic characteristics, design rational human dosing regimens and routes of administration. Finally, regarding the evaluation of efficacy, this study involves multiple behavioral tests and biochemical indicators as outcome measures. Future clinical trials can draw on these indicators and, in combination with clinical practice, select more sensitive and specific assessment tools to measure the symptom improvement of patients with depression.

## 5 Strengths

To our knowledge, the strength of this study lies in its being the first systematic review and meta-analysis of quercetin’s antidepressant effects in preclinical research. Through a comprehensive analysis of multiple behavioral tests and biochemical indicators, the ameliorative effects of quercetin on depressive-like symptoms in animal experiments were evaluated. Our findings provide practical value for translating animal data to clinical evidence.

## 6 Limitations

However, there are several limitations in our systematic review and meta-analysis. First, the number of studies included is limited, and the total sample size is relatively small. Second, the included studies lacked standardized protocols regarding animal species, depression modeling methods, treatment interventions, and outcome assessment methods, which led to higher heterogeneity. Third, most of the studies included did not implement effective allocation concealment measures, nor did they blind the researchers or outcome assessors, which may have introduced selection, performance, and detection biases. Finally, although the sensitivity analysis indicated that the results were relatively stable, the significant risk of bias remains a concern. Therefore, further high-quality studies are urgently needed to validate the results presented in this study.

Subgroup analyses revealed that the effects of quercetin on the total distance traveled in the open field test were significantly influenced by dose, treatment duration, and animal species. Specifically, high doses (≥60 mg/kg) and prolonged treatment durations (2–4 weeks) significantly increased the total distance traveled in rats. However, no significant effects were observed in mice or under conditions of low doses and short treatment durations. Collectively, these findings indicate that treatment duration, dosage, and animal species significantly influence the therapeutic efficacy of quercetin. Therefore, future studies should further standardize experimental protocols.

## 7 Conclusion

In this study, we conducted a Meta-analysis for the first time to demonstrate that quercetin exhibits significant antidepressant effects in animal studies. The underlying mechanisms may involve the regulation of oxidative stress, inflammatory responses, neurotrophic factors, and HPA axis function. Although the results are limited by the heterogeneity of animal models and the risk of bias, the multitarget properties of quercetin provide a theoretical basis for its potential as an antidepressant agent. Future research should focus on standardized preclinical studies and explore its clinical translation, including dose optimization, sex differences, and combination therapy strategies.

## Data Availability

The raw data supporting the conclusions of this article will be made available by the authors, without undue reservation.
