# Supplementary material for: Therapeutic potential of quercetin in depressive symptoms: a systematic review and meta-analysis of preclinical studies
Source: Front Pharmacol. 2025 Jul 8;16:1598053. doi: 10.3389/fphar.2025.1598053 (PMC12280726; doi:10.3389/fphar.2025.1598053)
Supplement: Supplementary file 1 [file DataSheet1.zip › Supplementary materials/Supplementary Table 1.docx]

**Supplementary Table 1** Subgroup analysis outcomes after quercetin intervention.

| **Outcome** | **Subgrouped by** | **NO. of studies** | **Heterogeneity** | | **SMD** | **95%CI** | ***P* value** |
| --- | --- | --- | --- | --- | --- | --- | --- |
|  |  |  | ***I²*（%）** | ***P* value** |  |  |  |
| **Immobility time in the FST** | **Duration (week)** |  |  |  |  |  |  |
|  | ≤1 | 7 | 49 | 0.07 | -4.58 | -5.96, -3.20 | ＜0.001 |
|  | ≤2 | 8 | 80 | ＜0.001 | -2.49 | -3.60, -1.38 | ＜0.001 |
|  | 2-4 | 9 | 77 | ＜0.001 | -2.26 | -3.28, -1.24 | ＜0.001 |
|  | ＞4 | 5 | 16 | 0.31 | -1.87 | -2.47, -1.26 | ＜0.001 |
|  | **Dose (mg/kg)** |  |  |  |  |  |  |
|  | ≤10 | 2 | 0 | 0.48 | -2.60 | -3.84, -1.35 | ＜0.001 |
|  | 10-60 | 17 | 71 | ＜0.001 | -2.27 | -2.93, -1.61 | ＜0.001 |
|  | ≥60 | 10 | 82 | ＜0.001 | -3.56 | -4.85, -2.27 | ＜0.001 |
|  | **Species** |  |  |  |  |  |  |
|  | Rats | 12 | 66 | ＜0.001 | -2.72 | -3.46, -1.98 | ＜0.001 |
|  | Outbred mice | 9 | 78 | ＜0.001 | -3.14 | -4.44, -1.84 | ＜0.001 |
|  | Inbred Mice | 7 | 75 | ＜0.001 | -1.89 | -2.94, -0.84 | ＜0.001 |
|  | Rare/Unspecified strains | 1 | — | — | -5.13 | -7.88, -2.38 | ＜0.001 |
| **Swimming time in the FST** | **Duration (week)** |  |  |  |  |  |  |
|  | ≤1 | 3 | 0 | 0.59 | 3.88 | 2.64, 5.13 | ＜0.001 |
|  | ≤2 | 1 | — | — | 7.68 | 4.46, 10.90 | ＜0.001 |
|  | 2-4 | 2 | 4 | 0.31 | 2.52 | 1.26, 3.77 | ＜0.001 |
|  | ＞4 | 0 |  |  |  |  |  |
|  | **Dose (mg/kg)** |  |  |  |  |  |  |
|  | ≤10 | 1 | — | — | 3.23 | 1.38, 5.07 | ＜0.001 |
|  | 10-60 | 2 | 71 | 0.06 | 3.23 | 0.36, 6.10 | 0.03 |
|  | ≥60 | 3 | 64 | 0.06 | 4.71 | 2.43, 6.99 | ＜0.001 |
|  | **Species** |  |  |  |  |  |  |
|  | Rats | 3 | 0 | 0.80 | 3.46 | 2.34, 4.58 | ＜0.001 |
|  | Outbred mice | 1 | — | — | 1.94 | 0.29, 3.59 | 0.02 |
|  | Inbred Mice | 2 | 42 | 0.19 | 6.13 | 3.42, 8.85 | ＜0.001 |
|  | Rare/Unspecified strains | 0 |  |  |  |  |  |
| **Total distance traveled in the OFT** | **Duration (week)** |  |  |  |  |  |  |
|  | ≤1 | 4 | 73 | 0.01 | 0.97 | -0.29, 2.24 | 0.13 |
|  | ≤2 | 2 | 0 | 0.40 | 0.08 | -0.56, 0.72 | 0.80 |
|  | 2-4 | 4 | 85 | ＜0.001 | 2.39 | 0.52, 4.25 | 0.01 |
|  | ＞4 | 1 | — | — | -0.28 | -1.13, 0.56 | 0.51 |
|  | **Dose (mg/kg)** |  |  |  |  |  |  |
|  | ≤10 | 2 | 93 | ＜0.001 | 1.44 | -3.04, 5.92 | 0.53 |
|  | 10-60 | 6 | 81 | ＜0.001 | 0.73 | -0.24, 1.70 | 0.14 |
|  | ≥60 | 3 | 59 | 0.09 | 1.88 | 0.63, 3.12 | 0.003 |
|  | **Species** |  |  |  |  |  |  |
|  | Rats | 9 | 83 | ＜0.001 | 1.44 | 0.46, 2.43 | 0.004 |
|  | Outbred mice | 1 | — | — | -0.17 | -1.05, 0.70 | 0.70 |
|  | Inbred Mice | 1 | — | — | -0.06 | -1.19, 1.07 | 0.92 |
|  | Rare/Unspecified strains | 0 |  |  |  |  |  |
| **Time spent in central area in the OFT** | **Duration (week)** |  |  |  |  |  |  |
|  | ≤1 | 3 | 79 | 0.009 | 5.24 | 1.72, 8.77 | 0.004 |
|  | ≤2 | 4 | 84 | ＜0.001 | 2.11 | 0.58, 3.64 | 0.007 |
|  | 2-4 | 3 | 80 | 0.007 | 1.40 | -0.27, 3.07 | 0.10 |
|  | ＞4 | 3 | 25 | 0.26 | 0.93 | 0.39, 1.47 | ＜0.001 |
|  | **Dose (mg/kg)** |  |  |  |  |  |  |
|  | ≤10 | 0 |  |  |  |  |  |
|  | 10-60 | 8 | 77 | ＜0.001 | 1.70 | 0.76, 2.63 | ＜0.001 |
|  | ≥60 | 5 | 85 | ＜0.001 | 2.31 | 0.87, 3.75 | 0.002 |
|  | **Species** |  |  |  |  |  |  |
|  | Rats | 6 | 78 | ＜0.001 | 2.25 | 0.99, 3.50 | ＜0.001 |
|  | Outbred mice | 4 | 80 | 0.002 | 1.80 | 0.32, 3.28 | 0.02 |
|  | Inbred Mice | 3 | 86 | 0.001 | 1.62 | 0.05, 3.19 | 0.04 |
|  | Rare/Unspecified strains | 0 |  |  |  |  |  |
| **Number of entries into central area in the OFT** | **Duration (week)** |  |  |  |  |  |  |
|  | ≤1 | 1 | — | — | 1.77 | 0.35, 3.20 | 0.01 |
|  | ≤2 | 0 |  |  |  |  |  |
|  | 2-4 | 4 | 72 | 0.01 | 1.13 | -0.01, 2.27 | 0.05 |
|  | ＞4 | 1 | — | — | 1.12 | 0.21, 2.04 | 0.02 |
|  | **Dose (mg/kg)** |  |  |  |  |  |  |
|  | ≤10 | 1 | — | — | 0.93 | -0.24, 2.10 | 0.12 |
|  | 10-60 | 4 | 20 | 0.29 | 0.86 | 0.25, 1.47 | 0.006 |
|  | ≥60 | 1 | — | — | 3.38 | 1.71, 5.04 | ＜0.001 |
|  | **Species** |  |  |  |  |  |  |
|  | Rats | 4 | 54 | 0.09 | 1.64 | 0.71, 2.57 | ＜0.001 |
|  | Outbred mice | 1 | — | — | 0.66 | -0.36, 1.67 | 0.20 |
|  | Inbred Mice | 1 | — | — | 0.11 | -1.02, 1.24 | 0.85 |
|  | Rare/Unspecified strains | 0 |  |  |  |  |  |
| **Number of standing episodes in the OFT** | **Duration (week)** |  |  |  |  |  |  |
|  | ≤1 | 2 | 87 | 0.006 | 2.38 | -1.36, 6.12 | 0.21 |
|  | ≤2 | 4 | 89 | ＜0.001 | -0.09 | -1.80, 1.63 | 0.92 |
|  | 2-4 | 2 | 0 | 0.80 | 1.91 | 1.00, 2.82 | ＜0.001 |
|  | ＞4 | 2 | 28 | 0.24 | 1.33 | 0.38, 2.27 | 0.006 |
|  | **Dose (mg/kg)** |  |  |  |  |  |  |
|  | ≤10 | 0 |  |  |  |  |  |
|  | 10-60 | 8 | 73 | ＜0.001 | 1.39 | 0.51, 2.26 | 0.002 |
|  | ≥60 | 2 | 91 | ＜0.001 | -0.72 | -3.32, 1.88 | 0.59 |
|  | **Species** |  |  |  |  |  |  |
|  | Rats | 6 | 85 | ＜0.001 | 0.63 | -0.58, 1.84 | 0.31 |
|  | Outbred mice | 4 | 86 | ＜0.001 | 1.63 | -0.34, 3.61 | 0.10 |
|  | Inbred Mice | 0 |  |  |  |  |  |
|  | Rare/Unspecified strains | 0 |  |  |  |  |  |
| **Immobility time in the TST** | **Duration (week)** |  |  |  |  |  |  |
|  | ≤1 | 4 | 77 | 0.005 | -2.21 | -3.90, -0.53 | 0.01 |
|  | ≤2 | 5 | 63 | 0.03 | -1.87 | -2.78, -0.96 | ＜0.001 |
|  | 2-4 | 8 | 83 | ＜0.001 | -3.05 | -4.62, -1.48 | ＜0.001 |
|  | ＞4 | 3 | 79 | 0.008 | -1.83 | -3.27, -0.38 | 0.01 |
|  | **Dose (mg/kg)** |  |  |  |  |  |  |
|  | ≤10 | 2 | 0 | 0.78 | -2.78 | -3.87, -1.69 | ＜0.001 |
|  | 10-60 | 11 | 78 | ＜0.001 | -2.40 | -3.38, -1.42 | ＜0.001 |
|  | ≥60 | 7 | 76 | ＜0.001 | -1.83 | -2.79, -0.87 | ＜0.001 |
|  | **Species** |  |  |  |  |  |  |
|  | Rats | 2 | 0 | 0.82 | -2.99 | -4.01, -1.97 | ＜0.001 |
|  | Outbred mice | 11 | 67 | ＜0.001 | -2.57 | -3.43, -1.71 | ＜0.001 |
|  | Inbred Mice | 7 | 75 | ＜0.001 | -1.36 | -2.26, -0.47 | 0.003 |
|  | Rare/Unspecified strains | 0 |  |  |  |  |  |
| **Sucrose preference in the SPT** | **Duration (week)** |  |  |  |  |  |  |
|  | ≤1 | 1 | — | — | 4.33 | 1.93, 6.73 | ＜0.001 |
|  | ≤2 | 5 | 50 | 0.09 | 1.57 | 0.88, 2.26 | ＜0.001 |
|  | 2-4 | 8 | 58 | 0.02 | 2.39 | 1.59, 3.19 | ＜0.001 |
|  | ＞4 | 7 | 83 | ＜0.001 | 1.48 | 0.57, 2.39 | 0.001 |
|  | **Dose (mg/kg)** |  |  |  |  |  |  |
|  | ≤10 | 2 | 0 | 0.40 | 4.35 | 2.86, 5.84 | ＜0.001 |
|  | 10-60 | 15 | 58 | 0.003 | 1.87 | 1.39, 2.35 | ＜0.001 |
|  | ≥60 | 4 | 80 | 0.002 | 1.15 | 0.09, 2.21 | 0.03 |
|  | **Species** |  |  |  |  |  |  |
|  | Rats | 10 | 49 | 0.04 | 2.08 | 1.54, 2.62 | ＜0.001 |
|  | Outbred mice | 6 | 78 | ＜0.001 | 1.85 | 0.76, 2.94 | ＜0.001 |
|  | Inbred Mice | 5 | 81 | ＜0.001 | 1.51 | 0.34, 2.68 | 0.01 |
|  | Rare/Unspecified strains | 0 |  |  |  |  |  |
| **Number of entries into open arms in the EPM** | **Duration (week)** |  |  |  |  |  |  |
|  | ≤1 | 2 | 0 | 0.76 | 1.03 | 0.15, 1.91 | 0.02 |
|  | ≤2 | 3 | 86 | ＜0.001 | 2.01 | -0.11, 4.13 | 0.06 |
|  | 2-4 | 2 | 95 | ＜0.001 | 2.80 | -2.01, 7.62 | 0.25 |
|  | ＞4 | 2 | 0 | 0.97 | 0.95 | 0.27, 1.63 | 0.006 |
|  | **Dose (mg/kg)** |  |  |  |  |  |  |
|  | ≤10 | 0 |  |  |  |  |  |
|  | 10-60 | 6 | 78 | ＜0.001 | 1.51 | 0.48, 2.54 | 0.004 |
|  | ≥60 | 3 | 85 | 0,001 | 1.86 | -0.17, 3.88 | 0.07 |
|  | **Species** |  |  |  |  |  |  |
|  | Rats | 4 | 84 | ＜0.001 | 2.11 | 0.52, 3.70 | 0.009 |
|  | Outbred mice | 2 | 0 | 0.96 | 0.92 | 0.13, 1.71 | 0.02 |
|  | Inbred Mice | 3 | 86 | ＜0.001 | 1.57 | -0.41, 3.56 | 0.12 |
|  | Rare/Unspecified strains | 0 |  |  |  |  |  |
| **Time spent in open arms in the EPM** | **Duration (week)** |  |  |  |  |  |  |
|  | ≤1 | 3 | 68 | 0.04 | 2.73 | 0.93, 4.52 | 0.003 |
|  | ≤2 | 4 | 91 | ＜0.001 | 4.57 | 0.73, 8.41 | 0.02 |
|  | 2-4 | 4 | 13 | 0.33 | 0.83 | 0.28, 1.38 | 0.003 |
|  | ＞4 | 4 | 0 | 0.79 | 0.64 | 0.25, 1.03 | 0.001 |
|  | **Dose (mg/kg)** |  |  |  |  |  |  |
|  | ≤10 | 0 |  |  |  |  |  |
|  | 10-60 | 10 | 64 | 0.003 | 1.15 | 0.53, 1.77 | ＜0.001 |
|  | ≥60 | 5 | 88 | ＜0.001 | 2.58 | 0.93, 4.23 | 0.002 |
|  | **Species** |  |  |  |  |  |  |
|  | Rats | 6 | 80 | ＜0.001 | 2.04 | 0.88, 3.21 | ＜0.001 |
|  | Outbred mice | 5 | 60 | 0.04 | 1.40 | 0.49, 2.31 | 0.002 |
|  | Inbred Mice | 4 | 84 | ＜0.001 | 1.19 | -0.14, 2.51 | 0.08 |
|  | Rare/Unspecified strains | 0 |  |  |  |  |  |
| **Time spent in closed arms in the EPM** | **Duration (week)** |  |  |  |  |  |  |
|  | ≤1 | 2 | 0 | 0.32 | -4.57 | -6.27, -2.88 | ＜0.001 |
|  | ≤2 | 0 |  |  |  |  |  |
|  | 2-4 | 2 | 59 | 0.12 | -1.23 | -2.32, -0.14 | 0.03 |
|  | ＞4 | 1 | — | — | -0.16 | -1.00, 0.68 | 0.71 |
|  | **Dose (mg/kg)** |  |  |  |  |  |  |
|  | ≤10 | 0 |  |  |  |  |  |
|  | 10-60 | 3 | 68 | 0.05 | -0.85 | -1.80, 0.11 | 0.08 |
|  | ≥60 | 2 | 0 | 0.32 | -4.57 | -6.27, -2.88 | ＜0.001 |
|  | **Species** |  |  |  |  |  |  |
|  | Rats | 4 | 87 | ＜0.001 | -2.51 | -4.44, -0.59 | 0.01 |
|  | Outbred mice | 1 | — | — | -0.66 | -1.68, 0.35 | 0.20 |
|  | Inbred Mice | 0 |  |  |  |  |  |
|  | Rare/Unspecified strains | 0 |  |  |  |  |  |
| **GSH** | **Duration (week)** |  |  |  |  |  |  |
|  | ≤1 | 1 | — | — | 2.17 | 0.43, 3.91 | 0.01 |
|  | ≤2 | 6 | 87 | ＜0.001 | 3.32 | 1.18, 5.46 | 0.002 |
|  | 2-4 | 5 | 82 | ＜0.001 | 3.74 | 1.99, 5.49 | ＜0.001 |
|  | ＞4 | 4 | 0 | 0.48 | 1.68 | 1.15, 2.20 | ＜0.001 |
|  | **Dose (mg/kg)** |  |  |  |  |  |  |
|  | ≤10 | 0 |  |  |  |  |  |
|  | 10-60 | 12 | 83 | ＜0.001 | 2.68 | 1.71, 3.64 | ＜0.001 |
|  | ≥60 | 4 | 0 | 0.43 | 3.36 | 2.34, 4.37 | ＜0.001 |
|  | **Species** |  |  |  |  |  |  |
|  | Rats | 10 | 77 | ＜0.001 | 3.32 | 2.30, 4.33 | ＜0.001 |
|  | Outbred mice | 5 | 80 | ＜0.001 | 1.75 | 0.34, 3.15 | 0.01 |
|  | Inbred Mice | 1 | — | — | 4.03 | 1.76, 6.30 | ＜0.001 |
|  | Rare/Unspecified strains | 0 |  |  |  |  |  |
| **SOD** | **Duration (week)** |  |  |  |  |  |  |
|  | ≤1 | 1 | — | — | 4.13 | 1.82, 6.44 | ＜0.001 |
|  | ≤2 | 5 | 87 | ＜0.001 | 2.49 | 0.44, 4.54 | 0.02 |
|  | 2-4 | 5 | 80 | ＜0.001 | 2.04 | 0.65, 3.43 | 0.004 |
|  | ＞4 | 3 | 83 | 0.003 | 3.37 | 1.30, 5.45 | 0.001 |
|  | **Dose (mg/kg)** |  |  |  |  |  |  |
|  | ≤10 | 0 |  |  |  |  |  |
|  | 10-60 | 12 | 83 | ＜0.001 | 2.58 | 1.55, 3.60 | ＜0.001 |
|  | ≥60 | 2 | 87 | 0.005 | 2.90 | -1.28, 7.08 | 0.17 |
|  | **Species** |  |  |  |  |  |  |
|  | Rats | 7 | 78 | ＜0.001 | 3.26 | 2.03, 4.49 | ＜0.001 |
|  | Outbred mice | 6 | 86 | ＜0.001 | 2.14 | 0.43, 3.85 | 0.01 |
|  | Inbred Mice | 1 | — | — | 0.93 | -0.29, 2.15 | 0.14 |
|  | Rare/Unspecified strains | 0 |  |  |  |  |  |
| **CAT** | **Duration (week)** |  |  |  |  |  |  |
|  | ≤1 | 1 | — | — | 2.13 | 0.59, 3.67 | 0.007 |
|  | ≤2 | 5 | 91 | ＜0.001 | 2.83 | 0.09, 5.57 | 0.04 |
|  | 2-4 | 4 | 73 | 0.01 | 2.20 | 0.83, 3.58 | 0.002 |
|  | ＞4 | 2 | 76 | 0.04 | 2.24 | 0.40, 4.07 | 0.02 |
|  | **Dose (mg/kg)** |  |  |  |  |  |  |
|  | ≤10 | 0 |  |  |  |  |  |
|  | 10-60 | 9 | 84 | ＜0.001 | 1.92 | 0.78, 3.07 | 0.001 |
|  | ≥60 | 3 | 56 | 0.10 | 3.90 | 1.92, 5.88 | ＜0.001 |
|  | **Species** |  |  |  |  |  |  |
|  | Rats | 5 | 91 | ＜0.001 | 2.23 | 0.13, 4.33 | 0.04 |
|  | Outbred mice | 5 | 0 | 0.63 | 2.58 | 1.84, 3.32 | ＜0.001 |
|  | Inbred Mice | 2 | 86 | 0.007 | 2.12 | -1.29, 5.53 | 0.22 |
|  | Rare/Unspecified strains | 0 |  |  |  |  |  |
| **MDA** | **Duration (week)** |  |  |  |  |  |  |
|  | ≤1 | 1 | — | — | -2.43 | -4.28, -0.59 | 0.01 |
|  | ≤2 | 5 | 21 | 0.28 | -2.17 | -2.94, -1.40 | ＜0.001 |
|  | 2-4 | 6 | 84 | ＜0.001 | -2.76 | -4.24, -1.29 | ＜0.001 |
|  | ＞4 | 2 | 60 | 0.11 | -2.33 | -3.74, -0.92 | 0.001 |
|  | **Dose (mg/kg)** |  |  |  |  |  |  |
|  | ≤10 | 0 |  |  |  |  |  |
|  | 10-60 | 11 | 74 | ＜0.001 | -2.60 | -3.43, -1.76 | ＜0.001 |
|  | ≥60 | 3 | 0 | 0.78 | -1.94 | -2.80, -1.08 | ＜0.001 |
|  | **Species** |  |  |  |  |  |  |
|  | Rats | 9 | 64 | 0.004 | -2.77 | -3.61, -1.94 | ＜0.001 |
|  | Outbred mice | 4 | 0 | 0.52 | -2.24 | -2.92, -1.55 | ＜0.001 |
|  | Inbred Mice | 1 | — | — | -0.07 | -1.20, 1.06 | 0.91 |
|  | Rare/Unspecified strains | 0 |  |  |  |  |  |
| **IL-6** | **Duration (week)** |  |  |  |  |  |  |
|  | ≤1 | 1 | — | — | -2.18 | -3.74, -0.63 | 0.006 |
|  | ≤2 | 7 | 83 | ＜0.001 | -2.92 | -4.51, -1.34 | ＜0.001 |
|  | 2-4 | 2 | 76 | 0.04 | -2.30 | -5.10, 0.50 | 0.11 |
|  | ＞4 | 1 | — | — | -2.44 | -3.66, -1.22 | ＜0.001 |
|  | **Dose (mg/kg)** |  |  |  |  |  |  |
|  | ≤10 | 0 |  |  |  |  |  |
|  | 10-60 | 6 | 65 | 0.01 | -1.70 | -2.62, -0.78 | ＜0.001 |
|  | ≥60 | 5 | 84 | ＜0.001 | -4.25 | -6.76, -1.73 | ＜0.001 |
|  | **Species** |  |  |  |  |  |  |
|  | Rats | 4 | 83 | ＜0.001 | -2.23 | -4.01, -0.46 | 0.01 |
|  | Outbred mice | 3 | 60 | 0.08 | -1.50 | -2.79, -0.21 | 0.02 |
|  | Inbred Mice | 2 | 82 | 0.02 | -4.45 | -9.17, 0.27 | 0.06 |
|  | Rare/Unspecified strains | 2 | 80 | 0.02 | -4.02 | -8.11, 0.07 | 0.05 |
| **TNF-α** | **Duration (week)** |  |  |  |  |  |  |
|  | ≤1 | 1 | — | — | -3.93 | -6.16, -1.71 | ＜0.001 |
|  | ≤2 | 6 | 77 | ＜0.001 | -3.42 | -5.10, -1.74 | ＜0.001 |
|  | 2-4 | 3 | 84 | 0.002 | -3.06 | -5.43, -0.70 | 0.01 |
|  | ＞4 | 4 | 91 | ＜0.001 | -7.36 | -11.84, -2.88 | 0.001 |
|  | **Dose (mg/kg)** |  |  |  |  |  |  |
|  | ≤10 | 1 | — | — | -6.33 | -8.69, -3.98 | ＜0.001 |
|  | 10-60 | 8 | 84 | ＜0.001 | -4.37 | -6.07, -2.66 | ＜0.001 |
|  | ≥60 | 5 | 80 | ＜0.001 | -3.49 | -5.50, -1.48 | ＜0.001 |
|  | **Species** |  |  |  |  |  |  |
|  | Rats | 7 | 83 | ＜0.001 | -5.55 | -7.58, -3.53 | ＜0.001 |
|  | Outbred mice | 3 | 83 | 0.003 | -3.37 | -6.26, -0.49 | 0.02 |
|  | Inbred Mice | 3 | 79 | 0.009 | -2.45 | -4.64, -0.26 | 0.03 |
|  | Rare/Unspecified strains | 1 | — | — | -3.93 | -6.16, -1.71 | ＜0.001 |
| **IL-1β** | **Duration (week)** |  |  |  |  |  |  |
|  | ≤1 | 0 |  |  |  |  |  |
|  | ≤2 | 4 | 75 | 0.008 | -2.00 | -3.65, -0.35 | 0.02 |
|  | 2-4 | 2 | 87 | 0.006 | -1.40 | -3.46, 0.66 | 0.18 |
|  | ＞4 | 4 | 81 | 0.001 | -3.02 | -4.77, -1.26 | ＜0.001 |
|  | **Dose (mg/kg)** |  |  |  |  |  |  |
|  | ≤10 | 1 | — | — | -3.61 | -5.14, -2.09 | ＜0.001 |
|  | 10-60 | 6 | 76 | ＜0.001 | -1.88 | -2.93, -0.82 | ＜0.001 |
|  | ≥60 | 3 | 83 | 0.003 | -2.76 | -5.41, -0.12 | 0.04 |
|  | **Species** |  |  |  |  |  |  |
|  | Rats | 6 | 72 | 0.003 | -2.47 | -3.53, -1.41 | ＜0.001 |
|  | Outbred mice | 1 | — | — | -0.36 | -1.35, 0.63 | 0.47 |
|  | Inbred Mice | 3 | 83 | 0.003 | -2.76 | -5.41, -0.12 | 0.04 |
|  | Rare/Unspecified strains | 0 |  |  |  |  |  |
| **BDNF** | **Duration (week)** |  |  |  |  |  |  |
|  | ≤1 | 0 |  |  |  |  |  |
|  | ≤2 | 4 | 62 | 0.05 | 2.86 | 1.14, 4.57 | 0.001 |
|  | 2-4 | 3 | 69 | 0.04 | 1.61 | -1.46, 4.69 | 0.30 |
|  | ＞4 | 4 | 12 | 0.33 | 0.87 | 0.31, 1.43 | 0.002 |
|  | **Dose (mg/kg)** |  |  |  |  |  |  |
|  | ≤10 | 1 | — | — | 1.20 | 0.23, 2.17 | 0.02 |
|  | 10-60 | 5 | 43 | 0.13 | 0.69 | -0.13, 1.51 | 0.10 |
|  | ≥60 | 5 | 68 | 0.01 | 2.65 | 1.00, 4.30 | 0.002 |
|  | **Species** |  |  |  |  |  |  |
|  | Rats | 5 | 48 | 0.10 | 1.09 | 0.16, 2.01 | 0.02 |
|  | Outbred mice | 3 | 75 | 0.02 | 1.21 | -0.56, 2.97 | 0.18 |
|  | Inbred Mice | 2 | 0 | 0.44 | 1.49 | 0.53, 2.45 | 0.002 |
|  | Rare/Unspecified strains | 1 | — | — | 8.05 | 3.96, 12.13 | ＜0.001 |
| **CORT** | **Duration (week)** |  |  |  |  |  |  |
|  | ≤1 | 2 | 91 | 0.001 | -13.66 | -33.91, 6.60 | 0.19 |
|  | ≤2 | 2 | 54 | 0.14 | -2.59 | -3.95, -1.23 | ＜0.001 |
|  | 2-4 | 3 | 51 | 0.13 | -1.60 | -2.65, -0.56 | 0.003 |
|  | ＞4 | 2 | 69 | 0.07 | -1.24 | -2.61, 0.13 | 0.08 |
|  | **Dose (mg/kg)** |  |  |  |  |  |  |
|  | ≤10 | 0 |  |  |  |  |  |
|  | 10-60 | 5 | 78 | 0.001 | -1.50 | -2.83, -0.17 | 0.03 |
|  | ≥60 | 4 | 11 | 0.34 | -2.76 | -3.57, -1.96 | ＜0.001 |
|  | **Species** |  |  |  |  |  |  |
|  | Rats | 6 | 84 | ＜0.001 | -2.79 | -4.41, -1.17 | ＜0.001 |
|  | Outbred mice | 3 | 0 | 0.47 | -1.32 | -2.03, -0.62 | ＜0.001 |
|  | Inbred Mice | 0 |  |  |  |  |  |
|  | Rare/Unspecified strains | 0 |  |  |  |  |  |

Abbreviations: CI, credibility interval; SMD, Standardized Mean Difference; FST, forced swimming test; TST, tail suspension test; SPT, sucrose preference test; OFT, open field test; EPM, elevated plus maze; CORT, corticosterone; BDNF, brain-derived neurotrophic factor; CAT, catalase; MDA, malondialdehyde; SOD, superoxide dismutase; GSH, glutathione; IL-1β, interleukin-1β; IL-6, interleukin-6; TNF-α, tumor necrosis factor-α.
