# Supplementary figures and images for: Therapeutic potential of quercetin in depressive symptoms: a systematic review and meta-analysis of preclinical studies
Source: Front Pharmacol. 2025 Jul 8;16:1598053. doi: 10.3389/fphar.2025.1598053 (PMC12280726; doi:10.3389/fphar.2025.1598053)

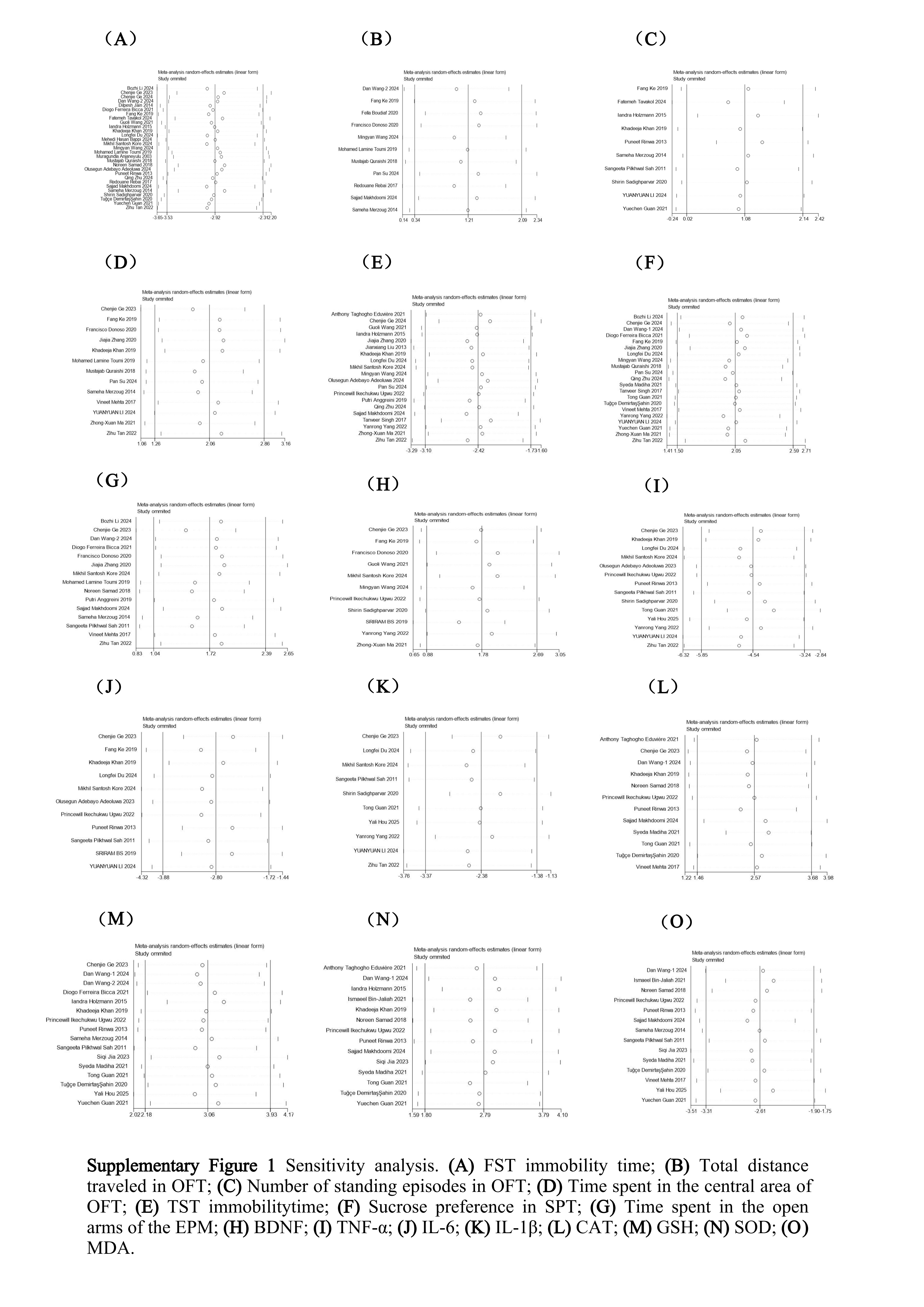

Supplement: Supplementary file 1 [file DataSheet1.zip › Supplementary materials/Supplementary Figure S1.tif]

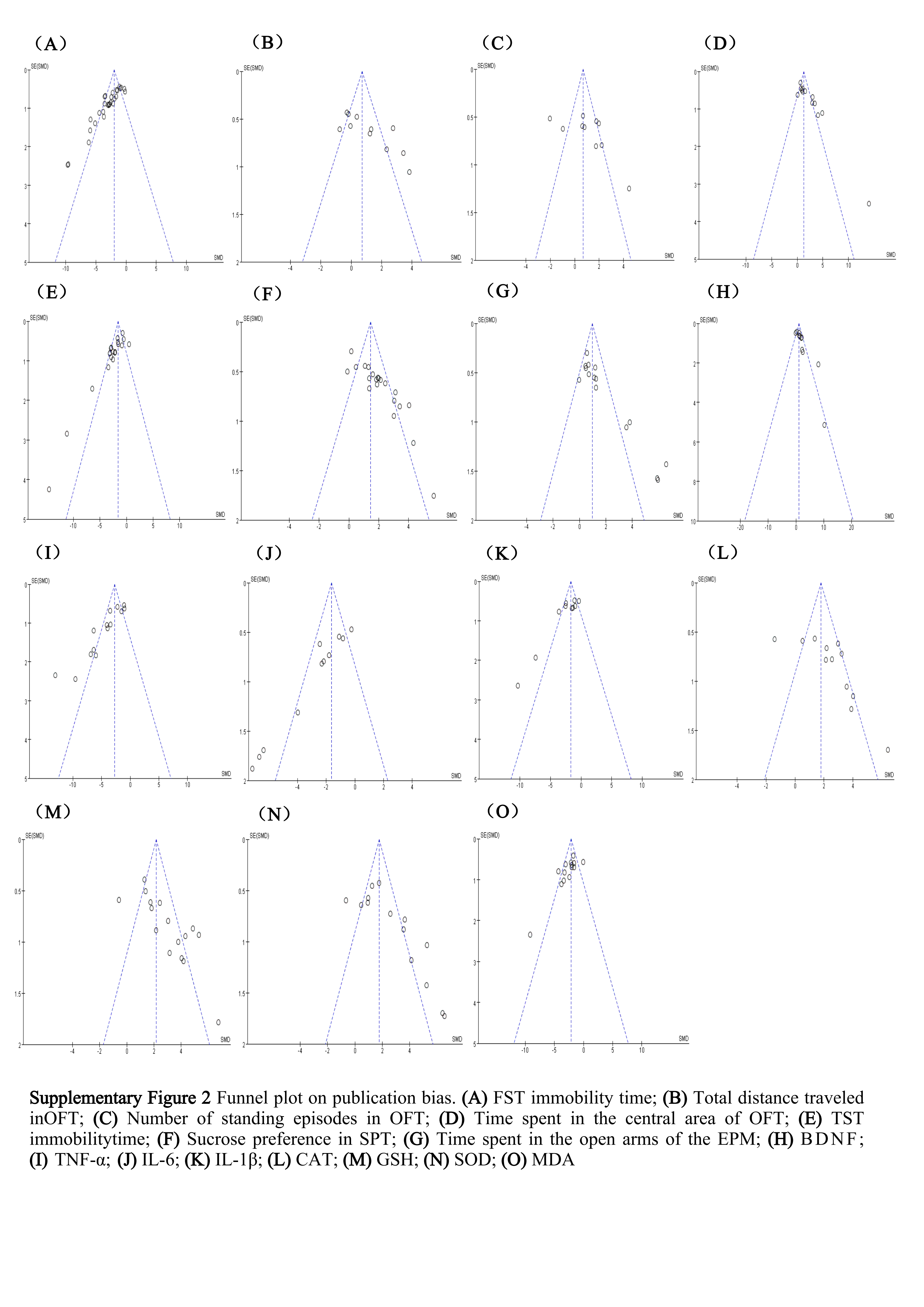

Supplement: Supplementary file 1 [file DataSheet1.zip › Supplementary materials/Supplementary Figure S2.tif]

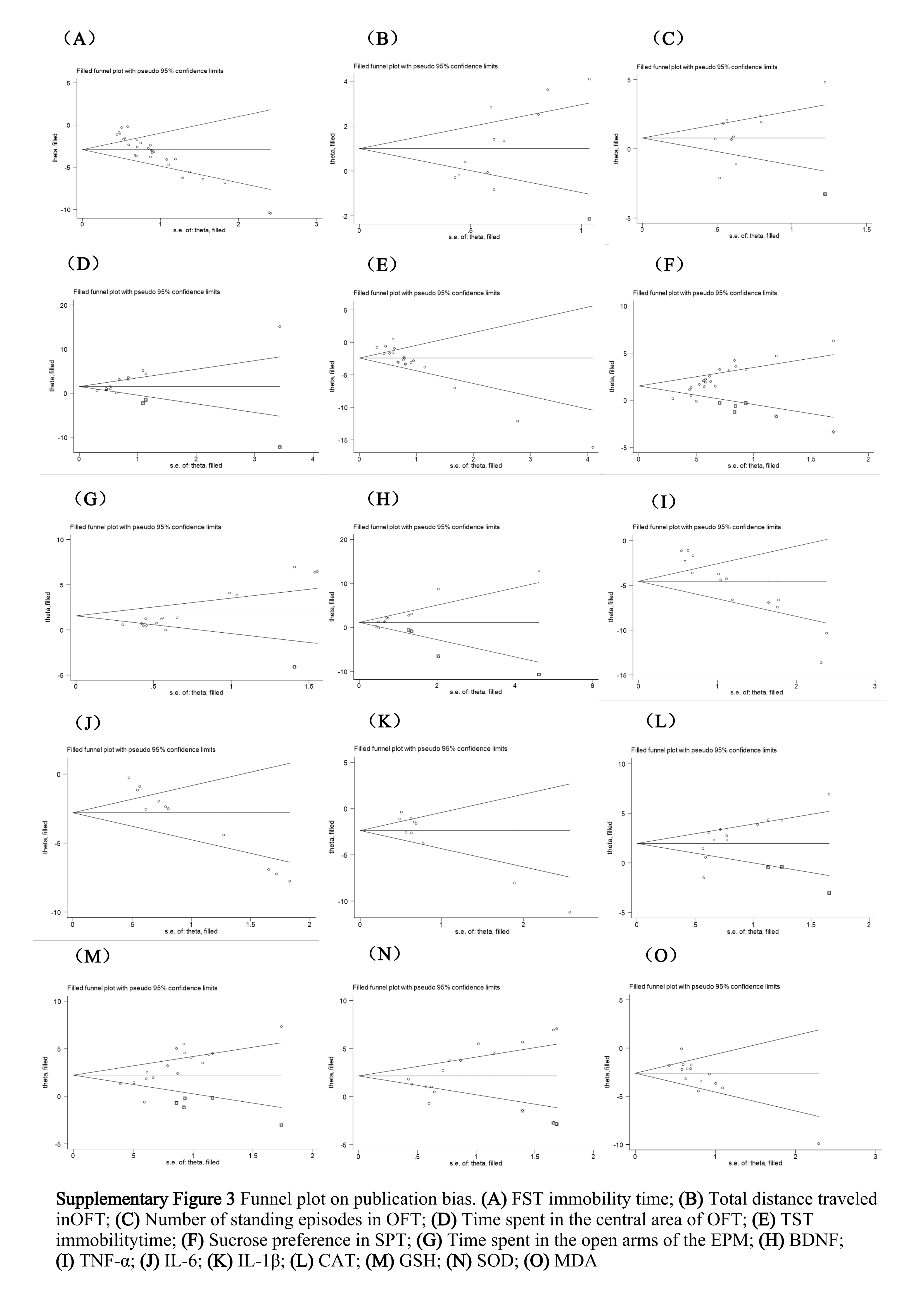

Supplement: Supplementary file 1 [file DataSheet1.zip › Supplementary materials/Supplementary Figure S3.tif]
